# Hepatic *Zbtb18* (Zinc Finger and BTB Domain Containing 18) alleviates hepatic steatohepatitis via *FXR* (Farnesoid X Receptor)

**DOI:** 10.1038/s41392-023-01727-7

**Published:** 2024-01-24

**Authors:** Lei Zhang, Jiabing Chen, Xiaoying Yang, Chuangpeng Shen, Jiawen Huang, Dong Zhang, Naihua Liu, Chaonan Liu, Yadi Zhong, Yingjian Chen, Kaijia Tang, Jingyi Guo, Tianqi Cui, Siwei Duan, Jiayu Li, Shangyi Huang, Huafeng Pan, Huabing Zhang, Xiaoqiang Tang, Yongsheng Chang, Yong Gao

**Affiliations:** 1 State Key Laboratory of Traditional Chinese Medicine Syndrome, Science and Technology Innovation Center, Guangzhou University of Chinese Medicine, Guangzhou, China; 2 Key Laboratory of Immune Microenvironment and Disease (Ministry of Education), Tianjin Key Laboratory of Cellular Homeostasis and Disease, Department of Physiology and Pathophysiology, Tianjin Medical University, Tianjin, China; 3 Jiangsu Key Laboratory of Immunity and Metabolism, Department of Pathogen Biology and Immunology, Jiangsu International Laboratory of Immunity and Metabolism, Xuzhou Medical University, Xuzhou, China; 4 Department of Endocrinology, The First Clinical College, Guangzhou University of Chinese Medicine, Guangdong, China; 5 The Fourth Clinical Medical College of Guangzhou University of Chinese Medicine, Shenzhen, China; 6 Jiangsu Collaborative Innovation Center of Traditional Chinese Medicine in Prevention and Treatment of Tumor, Nanjing University of Chinese Medicine, Nanjing, China; 7 Department of Biochemistry and Molecular Biology, Metabolic Disease Research Center, School of Basic Medicine, Anhui Medical University, Hefei, China; 8 Key Laboratory of Birth Defects and Related Diseases of Women and Children of MOE, State Key Laboratory of Biotherapy, West China Second University Hospital, Sichuan University, Chengdu, China

**Keywords:** Metabolic disorders, Inflammation

## Abstract

A lasting imbalance between fatty acid synthesis and consumption leads to non-alcoholic fatty liver disease (NAFLD), coupled with hepatitis and insulin resistance. Yet the details of the underlying mechanisms are not fully understood. Here, we unraveled that the expression of the transcription factor *Zbtb18* is markedly decreased in the livers of both patients and murine models of NAFLD. Hepatic *Zbtb18* knockout promoted NAFLD features like impaired energy expenditure and fatty acid oxidation (FAO), and induced insulin resistance. Conversely, hepatic *Zbtb18* overexpression alleviated hepato-steatosis, insulin resistance, and hyperglycemia in mice fed on a high-fat diet (HFD) or in diabetic mice. Notably, in vitro and in vivo mechanistic studies revealed that *Zbtb18* transcriptional activation of Farnesoid X receptor (*FXR*) mediated FAO and Clathrin Heavy Chain (*CLTC*) protein hinders NLRP3 inflammasome activity. This key mechanism by which hepatocyte’s *Zbtb18* expression alleviates NAFLD and consequent liver fibrosis was further verified by *FXR’*s deletion and forced expression in mice and cultured mouse primary hepatocytes (MPHs). Moreover, *CLTC* deletion significantly abrogated the hepatic *Zbtb18* overexpression-driven inhibition of NLRP3 inflammasome activity in macrophages. Altogether, *Zbtb18* transcriptionally activates the *FXR*-mediated FAO and *CLTC* expression, which inhibits NLRP3 inflammasome’s activity alleviating inflammatory stress and insulin resistance, representing an attractive remedy for hepatic steatosis and fibrosis.

## Introduction

Chronic or excessive lipids exposure resulting from prolonged high caloric intake frequently leads to steatohepatitis (steatosis with inflammation), liver fibrosis, cirrhosis, and eventually even hepatocellular carcinoma, representing the most relevant etiological factor of chronic liver diseases.^1–4^ Alarmingly, the incidence of lipid disorders in both adults and children is steadily rising due to the ongoing metabolic syndrome epidemic, which also entails obesity and diabetes.^5,6^

Mounting lines of evidence indicate that imbalances between intrinsic lipogenesis and FAO-derived lipid consumption contributes to lipotoxicity and NAFLD occurrence.^7–10^ In mammals, an abundant lipid deposition within the hepatocytes severely impairs the lysosomal-mitochondrial interaction in a vicious ROS-JNK feed-forward loop, eventually causing hepatocytes’ death.^11,12^ Moreover, lipotoxic injury stimulates liver macrophages accumulation and activates the NLRP3 inflammasome assembly and activation, driving the release of pro-inflammatory cytokines, such as TNF-α, IL-6 and IL-1β, providing a key pathogenetic link to nonalcoholic steatohepatitis (NASH) progression.^13,14^ Previous studies proved that pharmacological or genetic restoration of an impaired liver FAO alleviated steatohepatitis and hindered liver fibrosis progression by decreasing ROS overproduction or by reducing lipid peroxidation and NLRP3 inflammasome activity.^15^ However, the detailed molecular mechanisms causing steatohepatitis development remain incompletely understood and no effective or prospective therapeutic approaches to this highly prevalent disease are presently available.

Currently, the worthy and promising but limited therapeutic approaches to hepatic lipid disorders focus mainly on nuclear receptor remodeling.^16^ Among them, the activation of *PPARα* (peroxisome proliferator-activated receptor α) and *FXR* (farnesoid X receptor) have shown discrete effects.^17^ As a hepatic and intestinal highly expressed nuclear receptor, *FXR* is closely involved in bile acid metabolism and, as proven by a recent study, has organ-dependent different functions on lipid metabolism by remodeling NLRP3-mediated inflammasome activity.^18,19^ In NAFLD patients or mice models, the treatment with optocollic acid (OCA), an *FXR* agonist, exerted a set of relevant hepatic effects, decreasing TAGs (triacylglycerols) and inflammation, improving insulin sensitivity, and mitigating steatohepatitis and liver fibrosis.^20^ Deletion of hepatic *FXR* or its target genes predisposes mice to hyperlipemia and insulin resistance, quickly resulting in the acute NASH phenotype when fed on an HFD due to irreversible lipotoxicity, oxidative burden, and steatohepatitis.^21^ The stability and transcriptional activity of *FXR* might be regulated by endogenous proteins, such as SIRT6, and KLF16.^22^ Yet, the details about the involved molecular mechanisms are far from clear.

The *Zbtb18* (Zinc Finger and BTB Domain Containing 18) gene encodes a C2H2-type zinc finger protein and shares a subclass of conservative POK (POZ and Krüppel)/BTB domains. Previous reports revealed that *Zbtb18* interacts with *CtBP2* (C-Terminal Binding Protein 2) gene to promote glioblastoma malignancy.^23^ Recent studies also showed that *Zbtb18* repressed transcriptional programs closely linked to non-neuronal cell identity and glioblastoma subtypes, participating in neurodevelopmental diseases.^24^ However, it is obscure that whether *Zbtb18* participates the hepatic glucolipid metabolism.

In this study, we showed that the hepatic *Zbtb18* could activates *FXR* transcription, and subsequently accelerates the hepatic FAO, thereby preventing the onset and development of NAFLD. Moreover, the *Zbtb18/FXR* axis-stimulated *CLTC* protein expression remarkably inhibits NLRP3 inflammasome’s activity and alleviated liver inflammatory infiltrations and liver fibrosis. Therefore, the *Zbtb18/FXR* axis represents a novel candidate to target for the treatment of NAFLD and NASH.

## Results

### Hepatic *Zbtb18* down-regulation is closely related to the development of NAFLD

**Fig. 1:**
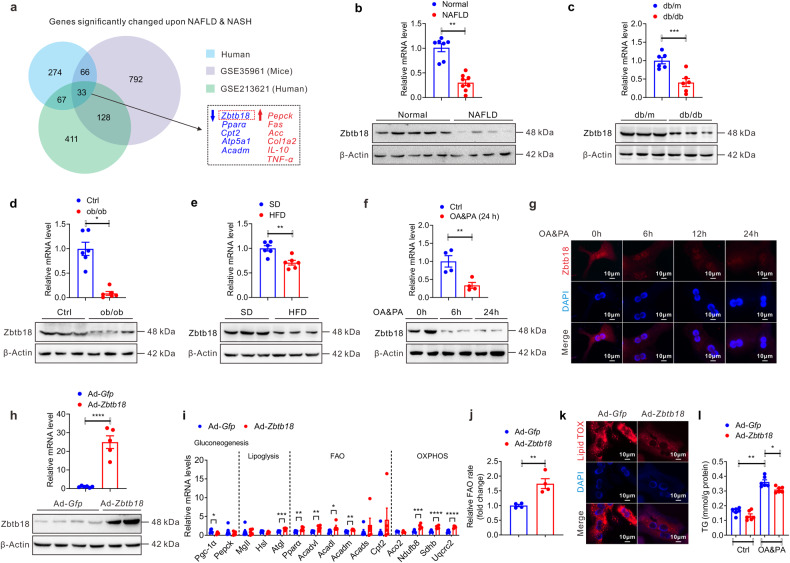
Hepatic *Zbtb18* mRNA and protein down-regulation is closely related to the development of NAFLD. **a** Venn diagram representing common significantly changed transcripts in liver from mice with NAFLD (GSE35961, *P* < 0.01), NAFLD patients (GSE213621, *P* < 0.05) and our clinic transcript data (*P* < 0.05). **b**–**e** Representative quantitative PCR and Western blot analysis of hepatic *Zbtb18* in clinical samples (**b**, normal controls = 7, NAFLD patients = 8); diabetic mice (**c**, *n* = 6); obese mice (**d**, *n* = 6); HFD-fed mice (**e**, *n* = 6). **f**, **g** Representative quantitative PCR, Western blot and immunofluorescence (IF) analysis of *Zbtb18* in MPHs treated with OA&PA; *n* = 4. **h** Representative quantitative PCR and Western blot data show an effective overexpression of *Zbtb18* in Ad-*Zbtb18* infected MPHs; *n* ≥ 5. **i** Representative quantitative PCR data show that *Zbtb18* overexpression significantly upregulates the genes involved in FAO and OXPHOS; *n* ≥ 5. **j** *Zbtb18* overexpression elevates the FAO rates in MPHs; *n* = 4. **k**, **l** *Zbtb18* overexpression decreases the lipid accumulation (**k**) and TGs contents (**l**) in MPHs; *n* = 6. Data are shown as means ± SEM. *P < 0.05; **P < 0.01; ***P < 0.005; ****P < 0.001

To achieve a systematic and comprehensive identification of key signaling molecules involved in the development of steatohepatitis, we first performed a mRNA microarray analysis of liver tissues from NAFLD patients and normal control, and subsequently crossed this clinic transcriptome data with published transcriptome data (GSE213621 from NAFLD patients and GSE35961 from mice model with NAFLD) to identify the differentially expressed genes (DEGs). The results exhibited there were 274, 411, and 792 DEGs from the 3 transcriptomes respectively. Additionally, 33 DEGs were overlapped among the three datasets. Of note, we found that hepatic *Zbtb18* expression was downregulated among these transcriptome data (Fig. 1a & Supplementary Fig. 1a–d). Furthermore, we confirmed the significant decrease in *Zbtb18* mRNA and protein in liver biopsies from NAFLD patients (Fig. 1b & Supplementary Table 1). Consistently, we also found the reduction of hepatic *Zbtb18* expression in several NAFLD mice models, including *db/db* diabetic mice, *ob/ob* obese mice, and HFD-induced obese mice (Fig. 1c–e). In addition, hepatic *Zbtb18* expression was markedly reduced in oleic acid & palmitic acid (OA & PA) cultured mouse primary hepatocytes (MPHs) (Fig. 1f–g). To preliminarily probe the physiological function of *Zbtb18*, we generated Ad-*Zbtb18*-infected MPHs and found that *Zbtb18* overexpression effectively increased the expression of genes involved in FAO and oxidative phosphorylation (OXPHOS), while it significantly suppressed the genes involved in gluconeogenesis (Fig. 1h, i). Accordingly, the *Zbtb18* overexpression increased fatty acid catabolism while decreasing lipid deposition in the MPHs (Fig. 1j–l). Therefore, our findings suggested that the hepatic *Zbtb18* was negatively related to the development and progression of NAFLD.

### Ablation of liver *Zbtb18* makes mice more prone to steatohepatitis

To explore the function of hepatic *Zbtb18*, we first generated hepatocyte-specific *Zbtb18* deletion (*Zbtb18*^*LKO*^) mice (Supplementary Fig. 2a). The *Zbtb18*^flox/flox^ mice were used as the control of *Zbtb18*^*LKO*^ mice. We found that *Zbtb18* deficiency led to severe lipid accumulation in cultured MPHs isolated from the *Zbtb18*^*LKO*^ mice (Supplementary Fig. 2b). Interestingly, hepatic *Zbtb18* deletion did not affect the body weight gain and energy expenditure of mice kept on a standard diet (SD) (Supplementary Fig. 2c, d). Yet, the blood glucose and serum insulin levels were increased in *Zbtb18*^*LKO*^ mice, even when fed on the SD (Supplementary Fig. 2e). Moreover, these mice displayed an impairment of glucose tolerance and insulin sensitivity, suggesting that hepatic *Zbtb18* was required to maintain glucose homeostasis (Supplementary Fig. 2f). Subsequent assays revealed that hepatic *Zbtb18*^*LKO*^ mice obviously increased the liver weight/body weight ratio value, and the circulating and hepatic TGs contents, which might lead to slight but visible lipid deposition in the liver (Supplementary Fig. 2g–i). Besides, *Zbtb18*^*LKO*^ mice exhibited increased serum ALT and AST levels (Supplementary Fig. 2j). In mechanistic terms, we preliminarily found that *Zbtb18* deletion suppressed the expression of genes involved in FAO and OXPHOS, which confirmed that *Zbtb18* is an important mediator of lipid catabolism in the liver (Supplementary Fig. 2k). Moreover, increased fatty acid oxidation by *Zbtb18* overexpression resulted in an increased serum ketone body, which is closely related to abnormal lipid deposition (Supplementary Fig. 2l).

**Fig. 2:**
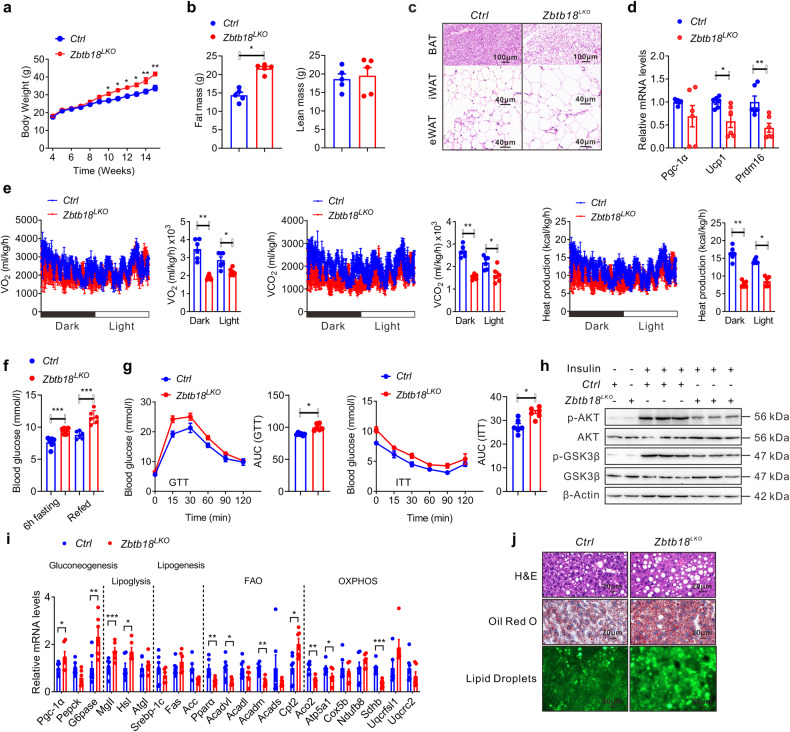
Hepatic ablation of *Zbtb18* aggravates steatohepatitis in mice fed on HFD. **a**, **b** Hepatic *Zbtb18* deletion leads to increases in body weight (**a**) and fat mass percentage (**b**) in mice fed on HFD; *n* ≥ 5. **c** Adipocyte hypertrophy of epididymal WAT, inguinal WAT and interscapular BAT of hepatic *Zbtb18* deleted mice fed on HFD. **d**, **e** Hepatic *Zbtb18* deletion decreases thermogenic genes (**d**) and energy expenditure (**e**) in BAT of mice fed on HFD; *n* ≥ 5. **f**, **g** Hepatic Zbtb*18* deletion increases the fasting blood glucose (**f**) and impairs glucose tolerance, and insulin sensitivity (**g**) in mice fed on HFD; *n* = 6. **h** Hepatic *Zbtb18* deficiency decreases the phosphorylation of AKT and GSK-3β in the livers of mice fed on HFD. **i**, **j** Hepatic *Zbtb18* deficiency alters the function of genes related to glucose and lipid metabolism (**i**) and leads to severe fatty liver phenotype (**j**) in mice fed on HFD, *n* = 6. Data are shown as means ± SEM. *P < 0.05; **P < 0.01; ***P < 0.005

Next, we prolongedly kept these mice on a high-fat diet (HFD) to further explore the physiological role of hepatic *Zbtb18* in lipid metabolism. We found that hepatic *Zbtb18* deletion easily predisposed the mice to obesity after a lengthy HFD exposure, as illustrated by their higher body weight and fat mass (Fig. 2a, b). Consistently, histological examination (H&E staining) also revealed an increased adipocyte hypertrophy of epididymal white adipose tissue (WAT), inguinal WAT, and interscapular brown adipose tissue (BAT) in *Zbtb18*^*LKO*^ mice (Fig. 2c). Correspondingly, these mice displayed a suppressed expression of thermogenic genes in the interscapular BAT, which might have contributed to their gain in body weight (Fig. 2d). Then, we housed the mice in metabolic cages and found that, compared to control mice, the *Zbtb18*^*LKO*^ mice showed lower degrees of energy expenditure, respiratory O_2_ consumption, and CO_2_ production (Fig. 2e). More importantly, *Zbtb18*^*LKO*^ mice showed an increase in fasting blood glucose and insulin levels in mice following HFD feeding (Fig. 2f & Supplementary Fig. 3a). Besides, *Zbtb18*^*LKO*^ mice exhibited an impairment of glucose tolerance and insulin sensitivity (Fig. 2g). Subsequent western blotting analyses also demonstrated the decrease in AKT and GSK-3β phosphorylation in livers from the *Zbtb18*^*LKO*^ mice, further suggesting the impaired insulin sensitivity in these mice (Fig. 2h). Moreover, hepatic *Zbtb18* deletion decreased the genes involved in FAO and OXPHOS, and up-regulated glycogenic genes, collectively increasing lipid deposition in the livers of mice fed on an HFD (Fig. 2i, j & Supplementary Fig. 3b, c). We also observed the decreased serum ketone body levels, increased serum TG and TC, coupled with elevation of serum pro-inflammatory cytokines levels in *Zbtb18*^*LKO*^ mice, which might be the result of the development and progression of NAFLD (Supplementary Fig. 3d–f).

### Hepatic *Zbtb18* overexpression safeguards from HFD-induced hepato-steatosis

**Fig. 3:**
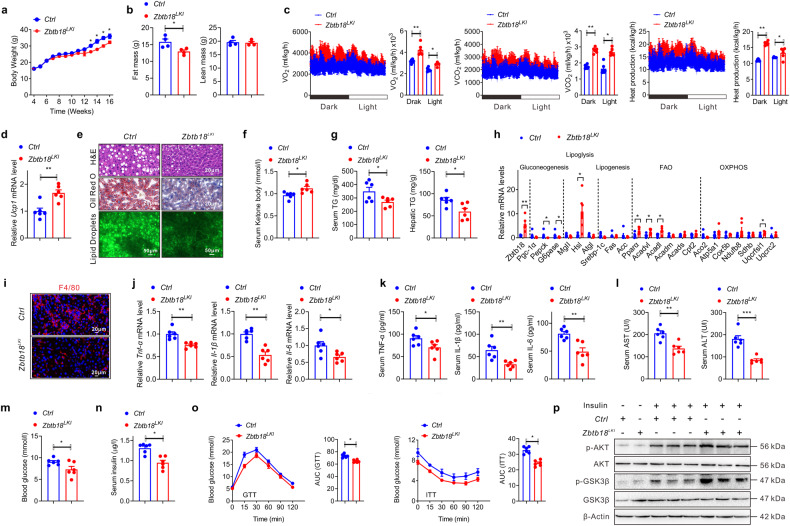
Hepatic *Zbtb18* overexpression defends against HFD-induced hepatic steatosis. **a**, **b** Hepatic *Zbtb18* overexpression decreases body weight (**a**) and fat mass (**b**) of mice fed on HFD; *n* ≥ 4. **c**, **d** Hepatic *Zbtb18* overexpression increases energy expenditure (**c**) and *Ucp1* expression in the BAT (**d**) of mice fed on HFD; *n* ≥ 5. **e**, **f** Hepatic *Zbtb18* overexpression improves the hepatic steatosis (**e**), increases serum ketone body levels (**f**) and decreases TGs contents in serum and livers of mice fed on HFD; *n* = 6. **g**, **h** Hepatic *Zbtb18* overexpression decreased serum and hepatic TG contents (**g**) and altered hepatic genes related to glucose and lipid metabolism (**h**) in mice fed on HFD; *n* ≥ 5. **i** Hepatic *Zbtb18* overexpression decreases F4/80^+^ cells in the livers of mice fed on HFD. **j**, **k** Hepatic *Zbtb18* overexpression decreases the mRNA levels of inflammatory genes (**j**) and serum proinflammatory cytokines levels (**k**) of mice fed on HFD; *n* = 6. **l** Hepatic *Zbtb18* overexpression decreases serum ALT and AST levels in mice fed on HFD; *n* = 6. **m**–**o** Hepatic *Zbtb18* overexpression decreases fasting blood glucose (**m**), and insulin levels (**n**) and improves glucose intolerance and insulin resistance (**o**) in mice fed on HFD; *n* = 6. **p** Hepatic *Zbtb18* overexpression enhances the phosphorylation of AKT and GSK-3β in the livers of mice fed on HFD. Data are shown as means ± SEM. *P < 0.05; **P < 0.01

To verify whether an increase in hepatic *Zbtb18* could improve the hepatic lipid and glucose homeostasis, we generated liver-specific *Zbtb18* overexpressing (*Zbtb18*^*LKI*^) mice via crossing Alb-Cre mice and Rosa26-*Zbtb18* mice (Supplementary Fig. 4a, b). The *ZTBT18*^*Rosa26*^ knock-in mice were used as the control of *Zbtb18*^*LKI*^. We first exposed these mice to HFD and found that the *Zbtb18* overexpression reduced body weight, which might ascribe to the decreases in fat mass and liver weight (Fig. 3a, b & Supplementary Fig. 4c). Moreover, compared to littermate control mice, *Zbtb18*^*LKI*^ exhibited enhanced energy expenditure, respiratory O_2_ consumption and CO_2_ production coupled with an increased *Ucp1* (Uncoupling protein 1) gene expression in BAT eventually contributing to the decrease in body weight (Fig. 3c, d). Of note, *Zbtb18* overexpression effectively alleviated HFD-induced hepatic steatosis, including the improvements in hepatocyte ballooning and lipid deposition (Fig. 3e). Besides, *Zbtb18*^*LKI*^ mice showed an increased serum ketone body levels and reduced hepatic and serum TGs levels (Fig. 3f, g). Correspondingly, *Zbtb18* overexpression increased the expression of genes involved in lipolysis, FAO, and OXPHOS, while genes related to lipogenesis were slightly changed (Fig. 3h). Furthermore, the improvement of excessive hepatic lipid accumulation effectively mitigated HFD-induced liver lipotoxicity. This alleviated the infiltration of inflammatory cells and the release of inflammatory cytokines, eventually reversing the hepatitis caused by HFD and improving liver function, which was reflected by reductions in the expression of inflammatory genes and serum ALT and AST levels (Fig. 3i–l). Consistently, due to the hepatic *Zbtb18* overexpression, these mice displayed lower fasting blood glucose and insulin levels (Fig. 3m, n). Hepatic *Zbtb18* overexpression also improved glucose tolerance and insulin sensitivity in the mice fed on HFD, and elevated the phosphorylation of AKT and GSK-3β in livers, suggesting a recovery from the altered insulin signaling due to HFD exposure (Fig. 3o, p). Moreover, the improvement of insulin sensitivity was further confirmed by the increase in phosphorylation of AKT and GSK-3β in MPHs after *Zbtb18* overexpression (Supplementary Fig. 5).

To determine the physiological function of hepatic *Zbtb18* in defending from glucose and lipid disorders, we next injected adeno-associated virus (AAV)-*Zbtb18* into hepatic *Zbtb18*^*LKO*^ mice to rescue hepatic *Zbtb18* expression. Thus, we found that a rescued expression of *Zbtb18* slightly decreased the body weight of the hepatic *Zbtb18*-deficient mice (Supplementary Fig. 6a). It also decreased the abnormal lipid deposition in the liver, as shown by the lowered serum and hepatic TG levels, and by an attenuation of the otherwise massive accumulations of large lipid droplets and of the ballooning degeneration of hepatocytes (Supplementary Fig. 6b, c). Besides, the fasting blood glucose and insulin levels were significantly decreased in the *Zbtb18*^*LKO*^ mice after AAV-*Zbtb18* injection (Supplementary Fig. 6d). Moreover, the hepatic *Zbtb18* rescue improved the glucose intolerance and insulin resistance of *Zbtb18*^*LKO*^ mice, also beneficially inducing enhanced phosphorylation of AKT and GSK-3β in *Zbtb18*-deficient liver (Supplementary Fig. 6e, f). Furthermore, the rescued expression of hepatic *Zbtb18* accelerated the liver FAO rate, as shown by the up-regulation of genes involved in FAO and OXPHOS, coupled with the increased serum ketone body levels (Supplementary Fig. 6g, h). Moreover, the rescued expression of hepatic *Zbtb18* significantly reduced the serum ALT and AST levels, indicating an improvement of hepatic function in these mice (Supplementary Fig. 6i). Collectively, these data proved that hepatic *Zbtb18* played an essential role in keeping the whole body’s glucose and lipid balance.

### Forced liver *Zbtb18* expression alleviates hepatic steatosis in diabetic mice

**Fig. 4:**
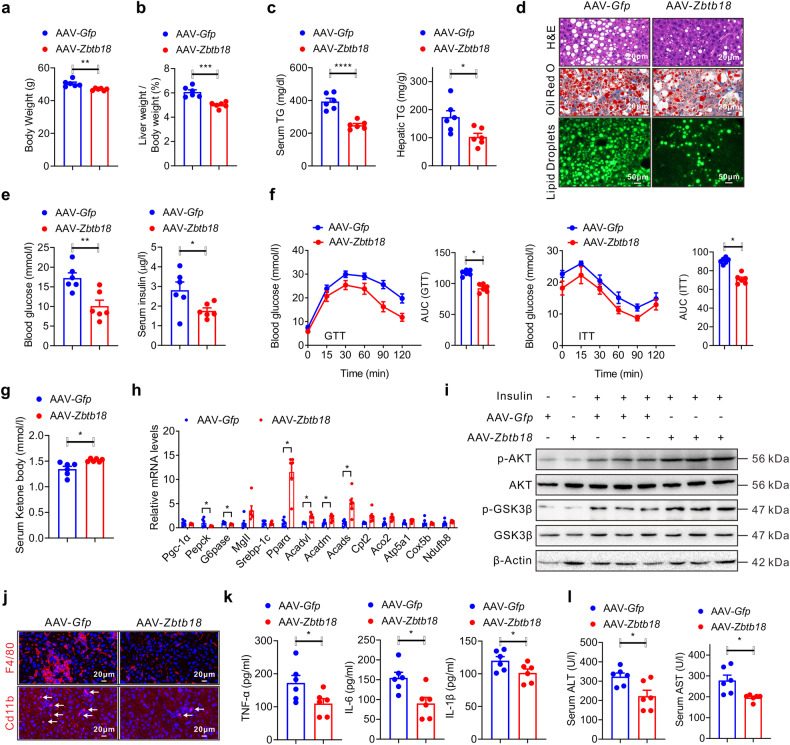
Rescued hepatic *Zbtb18* expression alleviates hepato-steatosis in diabetic mice. **a**–**c** Rescued hepatic *Zbtb18* expression decreases the body weight (**a**), the ratio values of liver weight to body weight (**b**) and TGs contents in the serum and livers (**c**) of *db/db* mice; *n* = 6. **d** AAV-*Zbtb18* infected *db/db* mice show an improved liver steatosis phenotype. **e**, **f** Hepatic *Zbtb18* overexpression significantly reduces fasting blood glucose and insulin levels (**e**), and improves glucose tolerance and insulin sensitivity (**f**) of *db/db* mice; *n* = 6. **g**, **h** Hepatic *Zbtb18* overexpression increases serum ketone body levels (**g**) and alters the expression of genes related to glucose and lipid metabolism (**h**) in *db/db* mice; *n* = 6. **i** Rescued *Zbtb18* expression in livers increases the phosphorylation of AKT and GSK-3β in *db/db* mice. **j** Hepatic *Zbtb18* overexpression decreases F4/80^+^ and Cd11b^+^ cells in the livers of *db/db* mice. **k**, **l** Hepatic *Zbtb18* overexpression reduces serum proinflammatory cytokines levels (**k**) and ALT, and AST levels (**l**) in *db/db* mice, *n* = 6. Data are shown as means ± SEM. *P < 0.05; **P < 0.01; ***P < 0.005; ****P < 0.001

Given the predominant role of hepatic *Zbtb18* in protecting diet-induced glucose and lipid disorders, we next tested whether its overexpression would improve the fatty liver phenotype in diabetic *db/db* mice. Thus, the hepatic *Zbtb18* expression was increased via tail vein injection with AAV-*Zbtb18* in *db/db* mice (Supplementary Fig. 7). Consistently, AAV-*Zbtb18*-infected *db/db* mice displayed reduced body weight and decreased liver weight/body weight ratio values (Fig. 4a, b). AAV-mediated hepatic *Zbtb18* overexpression also markedly improved the fatty liver phenotype in *db/db* mice, as revealed by the gross changes of TGs contents and by histological analysis (Fig. 4c, d). Likewise, the AAV-*Zbtb18* infected *db/db* mice showed decreased fasting blood glucose and insulin levels (Fig. 4e). The latter results implied an improvement of the glucose disorder in *db/db* mice, which was further confirmed by the ameliorated glucose intolerance and insulin resistance (Fig. 4f). Moreover, elevated serum ketone body levels were also noticed in AAV-*Zbtb18* injected *db/db* mice (Fig. 4g). Correspondingly, hepatic *Zbtb18* overexpression significantly increased the expression of genes involved in fatty acid oxidation, while glucogenic genes including *Pgc-1α* and *Pepck* were suppressed (Fig. 4h). A concurrently increased phosphorylation of AKT and GSK-3β in the liver, contributed to the recovery from insulin dysfunction in *db/db* mice (Fig. 4i). At the same time, *Zbtb18* overexpressing *db/db* mice showed significantly decreased gathering of hepatic F4/80 and Cd11b-positive macrophages within the liver, which together with the reduced inflammatory cytokines levels (Fig. 4j, k). All of these changes contributed to the improvement of hepatic dysfunction, as proven by the decreased serum ALT and AST levels in these mice (Fig. 4l). Altogether, these data indicated that targeting hepatic *Zbtb18* represents an effective approach to hinder the progression of liver steatosis in *db/db* mice.

### *Zbtb18* accelerates lipid catabolism via the transcriptional activation of *FXR*-mediated FAO

**Fig. 5:**
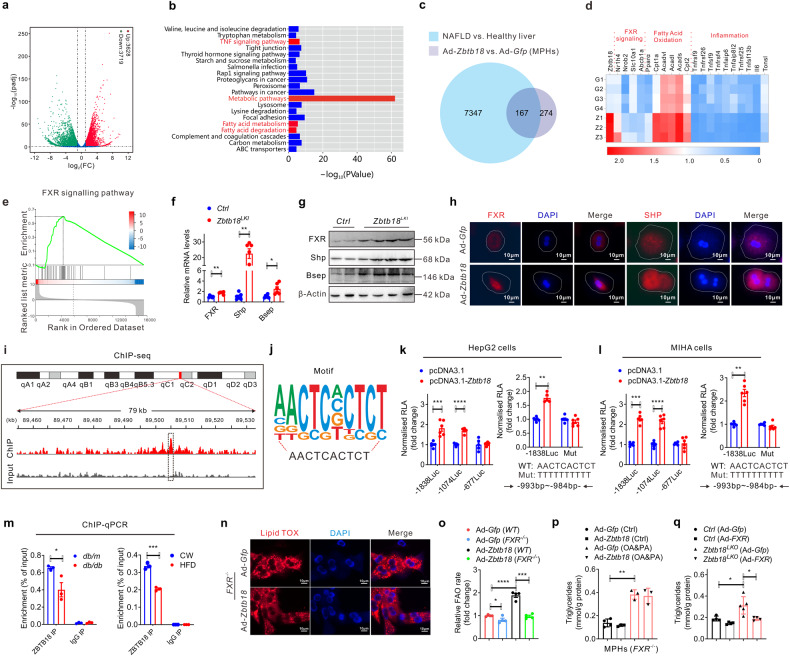
Zbtb18 transcriptional activation of *FXR* accelerates lipid catabolism via FAO. **a**, **b** RNA-seq data of *Zbtb18*-overexpressing and control MPHs show that *Zbtb18* alters the expression of genes related to FAO and the *FXR* signaling pathway, as shown by Scatter plot (**a**) and KEGG analysis (**b**). **c** Venn diagram showing the overlapping significantly differentially expressed transcripts identified in MPHs overexpressing *Zbtb18* (*P* < 0.01) and the liver form the NAFLD patients (*P* < 0.01). **d** Heatmap of transcriptome data showing the mRNA levels of genes involved in the FAO and *FXR* signaling pathway. **e** GSEA analysis of cellular components of DEGs identified in *Zbtb18*-overexpressing primary hepatocytes and the patients with NAFLD. **f**, **g** Representative quantitative PCR and Western blot analysis of *FXR* and of its target genes in hepatic *Zbtb18* overexpressing mice and control mice, *n* ≥ 5. **h** Immunofluorescence analysis reveals that *Zbtb18* overexpression increases *FXR* and *SHP* levels in the cytoplasm and nucleus of cultured MPHs. **i** Map of the *FXR* locus revealing *Zbtb18* binding in MPHs; aligned reads were visualized by Integrated Genomics Viewer 2. The signal of the IgG or Anti-Zbtb18 is represented with gray or red peaks, respectively. **j** The predicted *Zbtb18*-binding motif. **k**, **l** Luciferase assays indicate that *Zbtb18* protein binds to a unique site (−993 bp to −984 bp) to transcriptionally activate *FXR* expression, *n* ≥ 5. **m** ChIP-qPCR analyses of the *Zbtb18* protein occupancy on the *FXR* promoter; *n* = 3. **n** *FXR* deletion diminished the *Zbtb18* protein-driven protective effects on lipid accumulation. **o**, **p** *FXR* deletion reduces the stimulatory effects on FAO induced by *Zbtb18* protein overexpression; *n* = 4 (**o**) and TGs contents (**p**) in MPHs; *n* ≥ 3. **q** Forced expression of *FXR* counteracts the *Zbtb18*-deficiency-induced elevation of TGs contents in MPHs; *n* ≥ 4. Data are shown as means ± SEM. *P < 0.05; **P < 0.01; ***P < 0.005; ****P < 0.001

To investigate the details about the effectors or signaling nodes involved in *Zbtb18-*mediated protective effects of the liver, the RNA-seq was performed on the *Zbtb18* overexpressing MPHs. Thus, we found that 7347 genes were differently changed due to the *Zbtb18* overexpression, of which 3628 were up-regulated and 3719 were down-regulated (Fig. 5a). Subsequent Gene Ontology (GO) analysis indicated that *Zbtb18* overexpression along with an obvious significant altering of the hepatic inflammation and fatty acid metabolism pathway (Fig. 5b). To gain insight into the underlying mechanisms by which *Zbtb18* regulated hepatic fatty acid metabolism and inflammation, we analyzed the overlapping DEGs identified from the *Zbtb18* overexpressing MPHs and the liver from the NAFLD patients. Notably, the results showed that most overlapping DEGs were involved in the *FXR* target genes (Fig. 5c, d). Moreover, gene set enrichment analysis (GSEA) of the above DEGs identified in *Zbtb18* overexpressing MPHs and the liver from the NAFLD patients showed that “*FXR* signaling pathway” was positively correlated with *Zbtb18* expression, suggesting that the expression of these DEGs may be driven by *FXR*-dependent mechanisms (Fig. 5e). In addition, up-regulated *FXR* mRNA and protein levels were noticed in the *Zbtb18* overexpressing livers (Fig. 5f–g). Consistently, *Zbtb18* overexpression also significantly increased both of the total and nuclear levels of *FXR* protein and its target genes in cultured MPHs (Fig. 5h).

To further explore the underlying mechanisms by which *Zbtb18* activates *FXR*, *Zbtb18*-specific ChIP-seq was performed in MPHs. The result revealed a significant *Zbtb18*-binding promoter region in the *FXR* gene (Fig. 5i). Besides, we further analyzed the ChIP-seq data and confirmed that “AACTCACTCT”, which was located in the promoter region of *FXR*, was a key binding motif of *Zbtb18* (Fig. 5j). Then, the luciferase assays using a series of reported constructs (p*FXR*-1838, p*FXR*-1074, and p*FXR*-677) in HepG2 and MIHA cells. We found that *Zbtb18* markedly stimulated the transcriptional activity of p*FXR*-1838 and p*FXR*-1074, while p*FXR*-677 elicited no effects, indicating a potential binding site for the *Zbtb18* transcription factor from −1074 bp to −677 bp (Fig. 5k, l, left). More importantly, mutation of the AACTCACTCT in the promoter region of *FXR* to TTTTTTTTTT diminished the *Zbtb18*-induced stimulatory effects on *FXR* transcriptional activity, suggesting that its binding site is located in the region of *FXR* (Fig. 5k, l, right). Additionally, our ChIP-qPCR analysis revealed that the occupancy of *Zbtb18* transcription factor on *FXR’s* promoter was significantly decreased in the livers of *db/db* mice and HFD-fed mice, which further details the mechanism underlying *FXR* dysregulation in these mice (Fig. 5m). To further validate the key role of *FXR* in mediating the *Zbtb18*-induced protective effects, we generated *FXR*-knockout MPHs and found that *FXR* deletion significantly weakened the *Zbtb18*-derived protective effects on FAO leading to an unaltered lipid accumulation level in MPHs (Fig. 5n–p). Conversely, *FXR* overexpression alleviated the *Zbtb18* deficiency stimulated lipid deposition in MPHs (Fig. 5q). Collectively, these data implied that a strong correlation existed between *Zbtb18*-mediated transcriptional activation of *FXR* and hepatocellular lipid homeostasis.

### Hepatic *Zbtb18* inhibits NLRP3 inflammasome’s activation in macrophages via an *FXR*-mediated *CLTC* protein expression

**Fig. 6:**
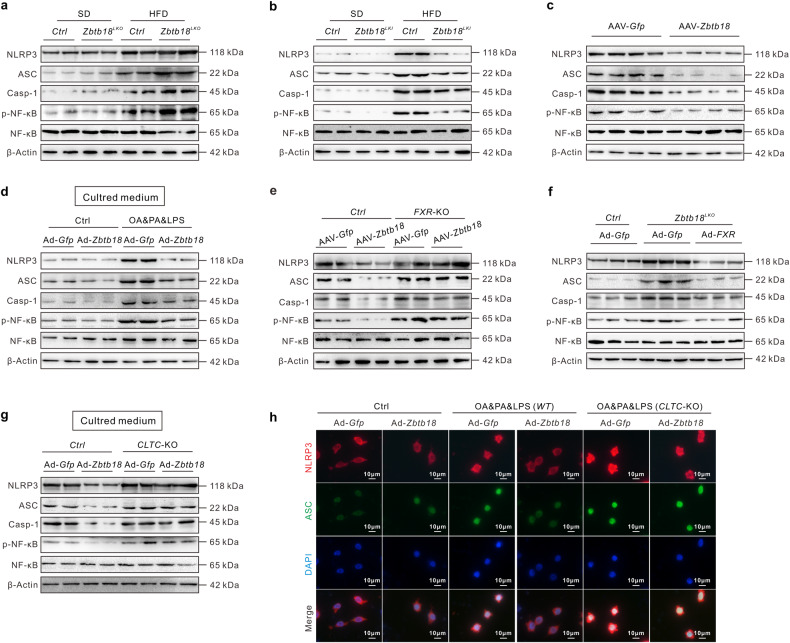
Hepatic *Zbtb18* protein inhibits NLRP3 inflammasome’s activation in macrophage via an *FXR*-mediated *CLTC* protein expression. **a** Hepatic *Zbtb18* deletion increases HFD-induced inflammasome-related proteins NLRP3, ASC, Caspase-1, and NF-кB phosphorylation in livers. **b** Hepatic *Zbtb18* overexpression decreases HFD-induced inflammasome-related proteins NLRP3, ASC, Caspase-1, and NF-кB phosphorylation in livers. **c** Hepatic *Zbtb18* overexpression decreases inflammasome-related proteins NLRP3, ASC, Caspase-1, and NF-кB phosphorylation in the livers of *db/db* mice. **d** Representative Western blot analyses indicated that the treatment with the conditioned medium of Ad-*Zbtb18* infected MPHs cultures suppressed the OA&PA&LPS-induced expression of inflammasome related proteins, NLRP3, ASC, Caspase-1, and NF-κb phosphorylation in BMDM macrophages. **e** *FXR* deletion diminishes the hepatic *Zbtb18* overexpression-induced protective effects on inflammasome-related proteins NLRP3, ASC, Caspase-1, and NF-кB phosphorylation in livers. **f** Hepatic *FXR* forced expression rescued the *Zbtb18* protein deficiency-induced expression of inflammasome-related proteins NLRP3, ASC, Caspase-1, and NF-кB phosphorylation in livers. **g**, **h** Representative Western blot and Immunofluorescence data show that the treatment with a conditioned medium of Ad-*Zbtb18*-infected *CLTC* knockout MPHs cultures fails to alter the OA&PA&LPS-induced expression of inflammasome related proteins NLRP3, ASC, Caspase-1, and NF-кB phosphorylation in BMDM cells

Emerging evidence suggests that a hepatocellular lipid imbalance always initiates and promotes an inflammation in the liver which in turn aggravates NAFLD progression.^25^ To comprehensively explore the impact of *Zbtb18* on NAFLD development, we also investigated the changes in NLRP3 inflammasome’s assembly and activation, which plays a key role in the liver inflammation in hepatic *Zbtb18* gain or loss models. Consistently, hepatic *Zbtb18* deletion substantially intensified the HFD-induced liver expression of the inflammasome-related proteins, including NLRP3, ASC, and Caspase-1. These effects were significantly reversed by a hepatic *Zbtb18* overexpression (Fig. 6a, b). Our results suggested a suppressing role of hepatic *Zbtb18* on NLRP3-mediated inflammasome activation, which was also verified in AAV-*Zbtb18* infected *db/db* mice (Fig. 6c). Subsequently, we performed the proteomic analysis of the supernatants of Ad-*Zbtb18* infected hepatocytes to uncover the key transmitter involved in the hepatocellular *Zbtb18*-driven suppression of inflammation. Thus, we found that *Zbtb18* overexpression significantly increased the abundance of proteins related to the inflammatory response, especially the *CLTC* protein’s amount, which is closely related to macrophage activity and potentially suppresses hepatic inflammation (Supplementary Fig. 8a–c), as previously reported.^26–28^ Subsequent experiments conducted on macrophages treated with the indicated cultured medium, also confirmed this assumption by showing that inflammasome’s activity was suppressed in macrophages cultured in Ad-*Zbtb18* infected hepatocytes conditioned medium (Fig. 6d). Interestingly, *FXR* deletion effectively abrogated the *Zbtb18*-elicited inhibition of NLRP3 inflammasome related proteins, while *FXR* overexpression effectively blocked the NLRP3 inflammasome’s abnormal activation in hepatic Zbtb*18-*deleted mice (Fig. 6e–f). Of note, the *Zbtb18*-stimulated *CLTC* protein expression was almost abolished by the *FXR* deletion in the hepatocytes and mice (Supplementary Fig. 8c, d). And deletion of *CLTC* in hepatocytes effectively diminished the inhibition of the NLRP3 inflammasome in macrophages induced by *Zbtb18* conditioned medium in vitro (Fig. 6g–h). Collectively, these results suggested that hepatic *Zbtb18* expression inhibited NLRP3 inflammasome’s activation in macrophages via an *FXR*-mediated *CLTC* protein expression, which helped mitigate NAFLD in mice.

### *FXR* activity effectively mediated the *Zbtb18*-induced attenuation of hepato-steatosis

**Fig. 7:**
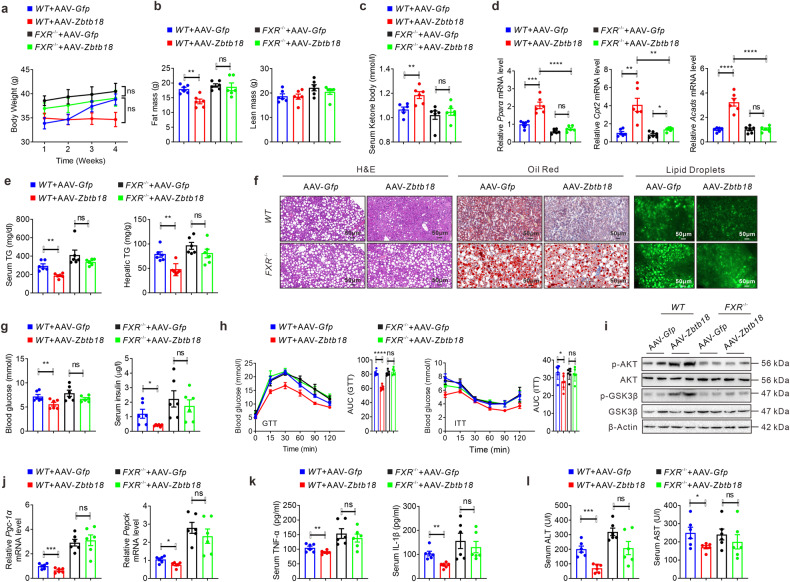
*FXR* ablation diminished hepatic *Zbtb18*-induced protective effects against steatohepatitis. **a**, **b** Hepatic *Zbtb18* overexpression fails to reduce the body weight (**a**) and fat mass (**b**) of *FXR* knockout mice; *n* = 6. **c**, **d** *FXR* deficiency abrogates the *Zbtb18* protein-stimulated elevation of serum ketone body (**c**) and expression of hepatic genes related to FAO (**d**); *n* = 6. **e**, **f** Hepatic *Zbtb18* overexpression fails to alter the TGs contents in the serum and liver (**e**), and the fatty liver phenotype (**f**) due to *FXR* deletion; *n* = 6. **g** Hepatic *Zbtb18* overexpression fails to change the fasting blood glucose and insulin levels in *FXR* knockout mice; *n* = 6. **h** The *Zbtb18*-driven improvement of glucose and insulin resistance was diminished in *FXR* knockout mice; *n* = 6. **i**–**j** Hepatic *Zbtb18* overexpression fails to change the phosphorylation of AKT and GSK-3β (**i**) and of glucogenic genes (**j**) in *FXR* knockout mice, *n* = 6. **k**, **l** Hepatic *Zbtb18* overexpression does not change serum proinflammatory cytokines (**k**) and ALT and AST levels (**l**) following *FXR* ablation; n = 6. Data are shown as means ± SEM. ns=no significant, *P < 0.05; **P < 0.01; ***P < 0.005; ****P < 0.001

In keeping with the above results, we tested the physiological function of *FXR* in mediating the *Zbtb18*-driven beneficial effects in vivo via tail vein injection of AAV-*Zbtb18* into *FXR*-deleted mice. Thus, we found that *FXR* deletion almost abolished the *Zbtb18*-induced reduction of body weight and fat mass, with rare changes in the energy expenditure in HFD mice (Fig. 7a, b & Supplementary Fig. 9a). Besides, the serum ketone body level was rarely changed in *FXR*-deleted mice after AAV-*Zbtb18* injection (Fig. 7c). Moreover, hepatic *Zbtb18* overexpression failed to alter the expression of hepatic genes involved in FAO (Fig. 7d & Supplementary Fig. 9b). This results in unchanged TGs contents in the serum and the livers of AAV-*Zbtb18* infected *FXR*-knockout mice, coupled with unchanged ballooning and lipid droplet accumulation in the hepatocytes (Fig. 7e, f & Supplementary Fig. 9c). Similarly, *FXR* deletion abrogated the decline of fasting blood glucose and insulin levels in serum after *Zbtb18* overexpression (Fig. 7g). In addition, the AAV-*Zbtb18* infected *FXR*-deficient mice showed no changes in glucose tolerance and insulin resistance (Fig. 7h). These results suggested a critical role of *FXR* in mediating hepatic *Zbtb18*-induced protective effects on glucose disorder. Notably, the *Zbtb18*-induced increase of phosphorylation of AKT and GSK-3β in livers was not observed in *FXR-*deleted mice (Fig. 7i). Also, no difference in the hepatic expression of glucogenic genes, such as *Pgc-1α* and *Pepck*, was detectable between AAV-*Zbtb18* infected mice and AAV-*Gfp* infected mice due to *FXR’s* deletion (Fig. 7j). Again, *Zbtb18* failed to reduce serum inflammatory cytokines, such as TNF-α and IL-1β, as well as ALT and AST levels in *FXR*-deficient mice (Fig. 7k–l).

**Fig. 8:**
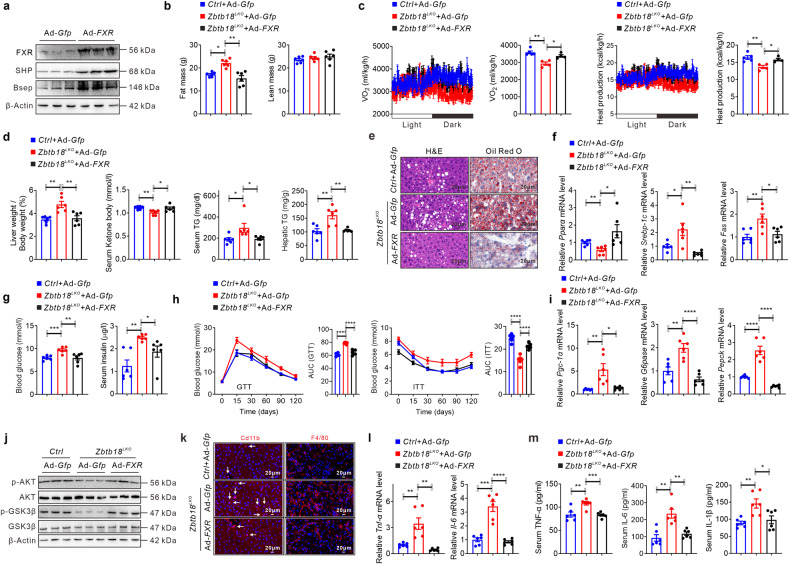
Hepatic *FXR* forced expression alleviates NAFLD phenotype in hepatic *Zbtb18* deleted mice. **a**, **b** Hepatic *FXR* overexpression (**a**) decreases the fat mass (**b**) of *Zbtb18*^*LKO*^ mice; *n* = 6. **c** Hepatic *FXR* overexpression rescues *Zbtb18* deficiency-induced impairment of energy expenditure; *n* ≥ 4. **d** Hepatic *FXR* overexpression decreases *Zbtb18* deficiency-induced liver weight gain and TGs accumulation in liver and serum, while recovering normal levels of serum ketone body; *n* = 6. **e**, **f** Hepatic *FXR* overexpression reduces *Zbtb18* deficiency-induced fatty liver phenotype (**e**), and the dysregulation of genes related to lipid metabolism (**f**); *n* = 6. **g** Hepatic *FXR* overexpression decreases the fasting blood glucose and insulin levels in *Zbtb18*^*LKO*^ mice; *n* = 6. **h** Hepatic *FXR* overexpression improves glucose and insulin resistance in *Zbtb18*^*LKO*^mice; *n* = 6. **i** Hepatic *FXR* overexpression decreases *Zbtb18* deficiency-induced glucogenic genes in mice; *n* = 6. **j** Hepatic *FXR* overexpression alleviates the impaired phosphorylation of AKT and GSK-3β in the livers of *Zbtb18*^*LKO*^ mice. **k** Hepatic *FXR* overexpression reduces the *Zbtb18* deficiency-induced gathering of F4/80^+^ and Cd11b^+^ cells in livers. **l**, **m** Hepatic *FXR* overexpression decreases the hepatic mRNA expression of inflammatory genes (**l**) and serum proinflammatory cytokines levels (**m**) in *Zbtb18*^*LKO*^ mice, *n* = 6. Data are shown as means ± SEM. *P < 0.05; **P < 0.01; ***P < 0.005; ****P < 0.001

On the contrary, *FXR*’s forced expression effectively increased the expression of its target genes in the livers of hepatic *Zbtb18*^*LKO*^ mice (Fig. 8a). These mice showed a lesser fat mass and enhanced energy expenditure due to *FXR* expression (Fig. 8b, c). Correspondingly, the changes in liver weight/body weight ratio values, serum ketone body levels, and TGs contents in hepatic *Zbtb18*^*LKO*^ mice were reversed due to the rescued activation of *FXR*, along with an improvement in the expression of hepatic genes involved in FAO and on the hepatic steatosis in these mice, as shown by the decreased ballooning due to lipid droplets accumulation in the hepatocytes (Fig. 8d–f). Moreover, increased *FXR* expression reduced fasting blood glucose and insulin levels in *Zbtb18*^*LKO*^ mice. Also, increased expression of *FXR* significantly alleviates glucose intolerance and insulin resistance in the liver caused by *Zbtb18*’s absence (Fig. 8g, h). Similar stimulatory effects on the phosphorylation of AKT and GSK-3β in livers were observed following *FXR* overexpression, indicating rescue of hepatic insulin signaling activity in *Zbtb18*^*LKO*^ mice, coupled with a beneficial regulation of the genes involved in glycogenesis (Fig. 8i, j). Of note, forced hepatic *FXR* expression effectively mitigated the *Zbtb18*-deficiency stimulated CD11b^+^ and F4/80^+^ macrophages gathering in livers, which attenuated the inflammatory stress and eventually contributed to the improvement of NAFLD in these mice (Fig. 8k–m). Collectively, these data strongly indicated a potential working scheme by which *Zbtb18*-mediated transcriptional activation of *FXR* preserved hepatic glucose and lipid homeostasis.

### Hepatic *Zbtb18* protein protects mice against MCD-induced liver fibrosis by transcriptionally activating *FXR*

**Fig. 9:**
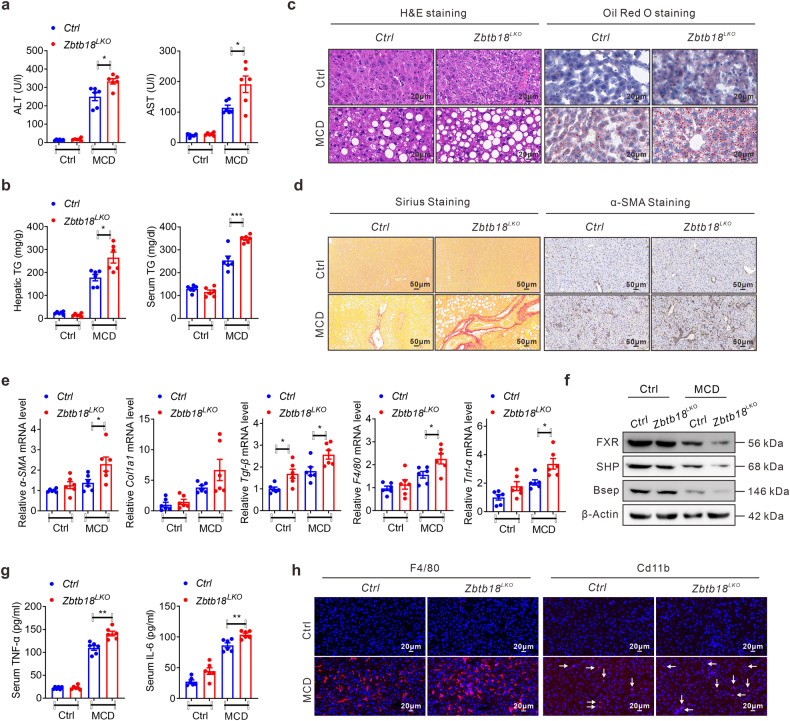
Hepatic *Zbtb18* deletion aggravates MCD-induced progression of liver fibrosis. **a**, **b** Serum ALT, and AST levels (**a**) and TGs contents in serum and livers (**b**) of hepatic *Zbtb18* deleted mice and control mice fed on MCD diet or on normal diet, *n* = 6. **c** Hepatic *Zbtb18* deletion aggravates MCD-induced liver injury and lipid deposition in livers. **d** Sirius and α-SMA staining of liver sections from hepatic *Zbtb18* deleted mice and control mice fed on MCD diet or a normal diet. **e** Hepatic *Zbtb18* deletion alters the expression of genes related to liver fibrosis and to an inflammatory response in the liver of mice fed on MCD diet or on a normal diet; *n* = 6. **f** Hepatic *Zbtb18* deletion aggravates the MCD-induced suppression of *FXR* and its downstream target genes in the liver. **g**, **h** Hepatic *Zbtb18* deletion aggravates the MCD-induced elevation of serum proinflammatory cytokines (**g**) and the hepatic gathering of F4/80^+^ and CD11b^+^cells (**h**); *n* = 6. Data are shown as means ± SEM. *P < 0.05; **P < 0.01; ***P < 0.005

Accumulating evidence suggests that a prolonged abnormal hepatic accumulation of lipids leads to excessive deposition of fibrous connective tissue and an imbalance of extracellular matrix synthesis and degradation in the liver, thereby advancing NASH progression.^29^ Considering the obvious effects of *Zbtb18* in decreasing hepatic lipid deposition, we suspected that it might exert a significant protective effect against liver fibrosis. Consequently, we first found that hepatic *Zbtb18* protein level was significantly decreased in (Methionine choline deficient) MCD diet fed mice (Supplementary Fig. 10a). Next, we fed *Zbtb18*^*LKO*^ mice with MCD diet to prove *Zbtb18*’s physiological function in the MCD diet induced development of liver fibrosis. As expected, in contrast to their littermate control mice, after MCD diet exposure *Zbtb18*^*LKO*^ mice had elevated ALT and AST levels (Fig. 9a), suggesting that a severe liver injury and hepatocytic death had occurred in these mice. Also, after MCD diet feeding, the TGs levels and hepatic lipid deposition were both aggravated due to *Zbtb18* deletion, as illustrated by the lipid droplet accumulation and consequent ballooning of the hepatocytes (Fig. 9b, c). Moreover, our Sirius Red staining analysis indicated that the MCD diet induced development of a fatal intraparenchymal pericellular fibrosis concurring with an acute elevation of α-SMA staining levels was promoted in hepatic *Zbtb18* ablated mice (Fig. 9d). Moreover, western blotting results also confirmed that *Zbtb18* deficiency accelerated the accumulation of α-SMA and Col1a1 in the liver (Supplementary Fig. 11a). Correspondingly, the MCD diet elicited expression of liver genes related to liver fibrosis development, including α-SMA, Col1a1, and TGF-β, was also stimulated in hepatic *Zbtb18*^*LKO*^ mice, along with an impaired expression of *FXR* and of its target genes (Fig. 9e, f). Moreover, hepatic *Zbtb18* deletion exacerbated the MCD diet induced inflammatory stress in the liver, as illustrated by the up-regulation of inflammatory genes, such as *F4/80* and TNF-α, and by the elevated levels of serum inflammatory cytokines, along with increased hepatic gathering of F4/80^+^ and CD11b+ macrophages (Fig. 9e, g, h). All of these events contribute to a fast steatohepatitis development.

**Fig. 10:**
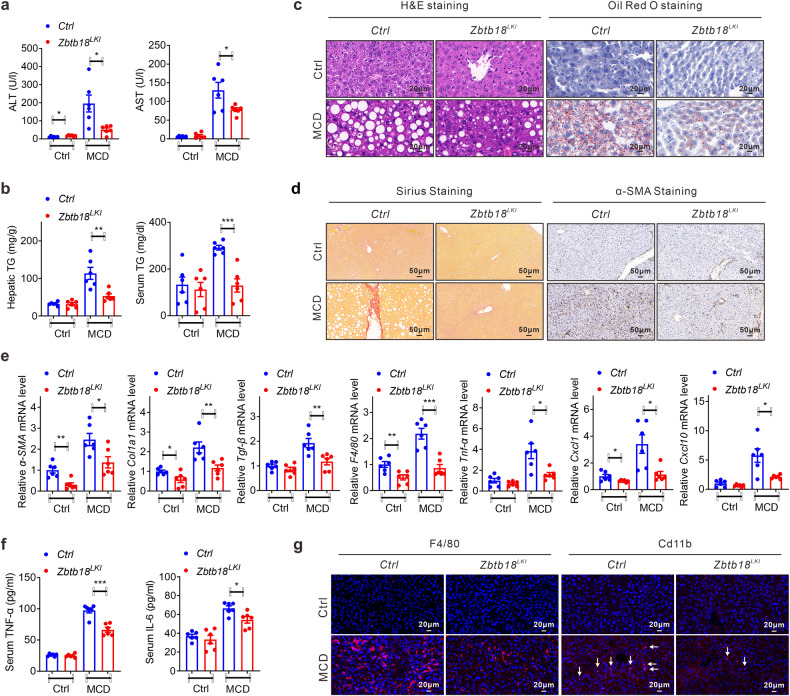
Hepatic *Zbtb18* overexpression protects mice against MCD-induced liver fibrosis. **a**, **b** Serum ALT, and AST levels (**a**) and TGs contents in serum and liver (**b**) samples from hepatic *Zbtb18* overexpressing mice and control mice fed on MCD diet or normal diet; *n* = 6. **c** Hepatic *Zbtb18* overexpression attenuates MCD-induced liver injury and hepato-steatosis. **d** Sirius and α-SMA staining of liver sections from hepatic *Zbtb18* transgenic mice and control mice fed on MCD diet or normal diet. **e** Hepatic *Zbtb18* overexpression alters the expression of genes related to liver inflammatory response and fibrosis in mice fed on MCD diet or normal diet; *n* = 6. **f**, **g** Hepatic *Zbtb18* overexpression reduces the MCD-induced elevation of serum proinflammatory cytokines (**f**) and the hepatic accumulation of F4/80^+^ and CD11b^+^ cells (**g**); *n* = 6. Data are shown as means ± SEM. *P < 0.05; **P < 0.01; ***P < 0.005

**Fig. 11:**
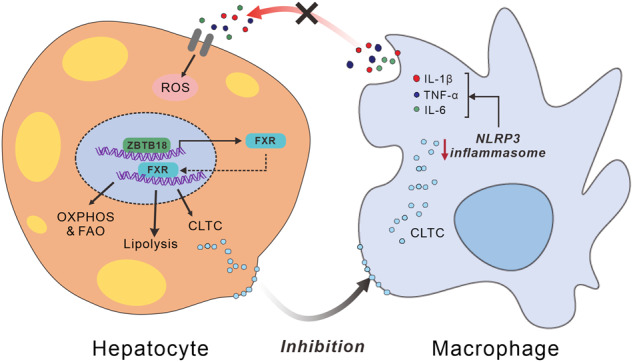
*Zbtb18* transcriptionally activates the *FXR*-mediated hepatic lipid metabolism, which inhibits NLRP3 inflammasome’s activity alleviating inflammatory stress and insulin resistance

By contrast, a hepatic *Zbtb18* overexpression protected mice against MCD diet induced liver fibrosis, as illustrated by the normal serum ALT and AST levels as compared to littermate control mice (Fig. 10a). Moreover, MCD diet stimulated TGs elevation and excess hepatic lipid deposition were effectively improved by *Zbtb18* overexpression, as shown by the H&E and Oil Red O staining analysis (Fig. 10b, c). Consistently, the Sirius staining and α-SMA immunohistochemistry analysis indicated that the development of intraparenchymal pericellular fibrosis was partly hindered in hepatic *Zbtb18* overexpressing mice, even when fed on MCD diet (Fig. 10d). Moreover, increased *Zbtb18* repressed the expression of a-SMA and Col1a1 in the liver (Supplementary Fig. 11b). This was coupled with a reduced expression of closely related genes, including α-SMA, Col1a1, and TGF-β (Fig. 10e). Moreover, hepatic *Zbtb18* overexpression suppressed the MCD diet induced inflammatory genes, such as F4/80, TNF-α, CXCL1, and CXCL10, in the liver, thereby reducing inflammatory cytokines’ serum levels and hepatic infiltration of inflammatory cells, helping improve the steatohepatitis (Fig. 10e–g). Collectively, these findings suggest a potentially therapeutic role of hepatic *Zbtb18* in defending against steatohepatitis partly via the activation of *FXR* and its downstream target genes (Fig. 11).

## Discussion

In mammals, the imbalance of de novo lipogenesis and FAO leads to a surplus hoarding of TGs in the hepatocytes, which contributes to the development of NAFLD, and even to its progression to NASH.^30^ As yet, the underlying causative mechanisms are far from clear. In the current study, we identified a close link between the hepatic dysregulation of *Zbtb18* and the onset and progression of NAFLD and NASH for the first time. Hepatic *Zbtb18* protein could transcriptionally activate *FXR* and its downstream target genes by directly binding to its promoter region: the upshot would be an accelerated FAO and consumption of excess lipids in the liver, helping improve steatohepatitis.

Accumulating evidence indicated that multiple especially endogenous factors, including environmental or physiological regulators, affect the lipogenesis and FAO in hepatocytes to keep lipid homeostasis.^31,32^ We and others previously found that cellular transcriptional factors, such as KLFs, which encode a variety of Krüppel-like factor subfamily of zinc finger proteins, are closely involved in the regulation of the hepatic glucose and lipid balance. *KLF16* and *KLF11* potentially bind to the promoter region of *PPARα* to activate FAO and improve the fatty liver phenotype.^33,34^ *KLF9*, a critical sensor of GRs, participates in fasting-induced hyperglycemia via the transcriptional activation of *Pgc-1α*.^35^ In this study, we found that the *Zbtb18*, another member of the transcriptional factors family (Zbtbs), which shares an alike C2H2-type Zinc finger and BTB Domain, is closely related to the development of NAFLD. As previously reported, ZBTBs play key roles in systemic metabolism homeostasis. *Zbtb11* cooperates with *NRF-2* to control mitochondrial function.^36^ *Zbtb20* acts as a key regulator of hepatic lipogenesis, influencing systemic lipid homeostasis.^37^ Likewise, we found that *Zbtb18* played effective roles in stimulating FAO and defending against the diet-induced NAFLD phenotype. Hepatic *Zbtb18* deletion severely hinders FAO in the liver while dramatically suppressing the genes involved in FAO, resulting in a conspicuous hepato-steatosis. By contrast, hepatic *Zbtb18* overexpression or rescue protects mice against HFD-induced fatty liver due to its stimulatory effects on fatty acid catabolism and FAO. Besides, chronic HFD exposure often fires “multiple hits” in the liver, resulting in impaired insulin response cascades and leading to insulin resistance, which has become an important complication and diagnostic index of NAFLD.^33^ In this regard, after *Zbtb18* overexpression we also found a glucose intolerance improvement and a reduced insulin resistance, coupled with an enhanced phosphorylation of AKT and GSK-3β in the liver. Since hepatic *Zbtb18* deletion predisposes HFD-fed mice to serious glucose intolerance and insulin resistance, it is easy to take it as an indication that *Zbtb18* plays a predominant role in alleviating the HFD-induced dysfunctional glucose and insulin responses.

Several studies have reported a Zbtbs-driven activation of *ChREBP-α* and *PCK1* in mediating the regulation of glucose and lipid metabolism by Zbtb20 or Zbtb16.^37,38^ Yet, we didn’t find alike changes after altering *Zbtb18* expression. In the present work, we subjected hepatocytes overexpressing the *Zbtb18* to RNA-seq to systematically explore the potential targets of the *Zbtb18*-mediated beneficial effects. Thus, we found that *Zbtb18* protein could effectively stimulate *FXR* expression and hence its downstream target genes. As previously reported, *FXR* protein, a highly expressed hepatic and intestinal nuclear receptor, works as a key regulator of bile acid metabolism in mammals and participates in the upkeeping of whole-body glucose and lipid homeostasis in an organ-dependent working fashion.^39^ We and others showed that increasing by pharmacological or genetic means the transcriptional activity or stability of the *FXR* protein exerted remarkably protective effects on inflammatory stress and acute damage in the liver. *FXR* activation by hepatocellular Cystathionine γ lyase/H_2_S significantly alleviates the diet-induced NAFLD, while iron-induced *FXR* suppression leads to fatal hepatotoxicity in humans and mice.^40^ In this work, we found that *Zbtb18* overexpression remarkably increased the *FXR* mRNA and protein levels and activated its target genes in livers and cultured MPHs by enhancing the nuclear-translocation of the *FXR* protein. These results suggest a transcriptional modulation of *FXR* by the *Zbtb18*. Subsequent luciferase assays and mutation analysis indicate the potential binding site for *Zbtb18* transcription factor on the −800bp to −789bp promoter region of *FXR*, a finding subsequently validated by ChIP and ChIP-qPCR data. The latter also complements and perfects the mechanism underlying *FXR* dysfunction, which senses and responds to a surplus lipid deposition. To prove *FXR’s* critical role in mediating the *Zbtb18*-induced protective effects against lipid and glucose metabolic disorders, we employed *FXR* gain or loss cultured MPHs and HFD-fed mice combining them with hepatic *Zbtb18* alterations. Consistently, we found that *FXR* deletion almost completely diminished the *Zbtb18*-induced beneficial effects on lipid deposition and glucose metabolism dysfunction in vivo and in vitro, coupled with a lack of effects on insulin resistance. Since *FXR* exerts different effects on lipid metabolism, including lipogenesis, FAO, and lipid absorption according to the anatomic location considered, we next injected Ad-*FXR* into hepatic *Zbtb18* deleted mice to monitor its effects on the glucose and lipid metabolism while suppressing *FXR*’s function in other tissues. Consistent with our hypothesis, the activation of hepatic *FXR* effectively offset the NAFLD phenotype and insulin resistance induced by *Zbtb18* silencing, suggesting a novel yet essential role for *FXR* as the mediator of hepatic *Zbtb18*’s key functions in maintaining glucose and lipid balance.

Several works have confirmed that a prolonged lipid overload always stimulates the gathering of macrophages inside the liver, resulting in chronic inflammation, which via the release of inflammatory cytokines, such as TNF-α, IL-6, and IL-1β, promotes NAFLD progression.^41,42^ As a consequence, the abnormal inflammatory stress destroys the balance between extracellular matrix synthesis and degradation, which leads to a surplus deposition of fibrous connective tissue in the liver, and NASH.^43^ Previous studies reported effective approaches preventing NASH development by improving the impaired hepatic FAO and the abnormal inflammatory response proper of the advanced stages of NAFLD.^44^ Functioning as critical transcriptional factors, Zbtbs were proven to be involved in the pathogenesis of many inflammatory disorders by flexibly modulating the development, differentiation, distribution, and effectors activity of immune cells.^45^ Our present results showed for the first time the critical role of hepatic *Zbtb18* in blocking NLRP3 inflammasome activity via an *FXR*-regulated *CLTC* protein expression, while hepatic *Zbtb18* deletion accelerated the gathering of F4/80^+^ and CD11b^+^ macrophages in the liver, leading to an increased expression of inflammatory genes and of circulating proinflammatory cytokines.

On the contrary, hepatic *Zbtb18* overexpression exerted opposite effects on HFD-induced hepatic inflammatory response partly via *FXR* activation, as illustrated by the decreased gathering of F4/80^+^ and CD11b^+^ macrophages in the liver, and by the suppression of inflammatory genes and of the release of proinflammatory cytokines in these mice. Subsequent MCD exposure experiments also revealed the vital role of *Zbtb18* in alleviating the development of steatohepatitis, as shown by the more intense hepatocytic death and liver injury that occurred in hepatic *Zbtb18* deleted mice. Conversely, hepatic *Zbtb18* overexpression protected the mice against MCD-induced intraparenchymal pericellular fibrosis by activating *FXR* and its downstream target genes. Altogether, our findings may explain the complex mechanisms by which *FXR* was dysregulated during the progress of liver fibrosis.

In summary, we proved that the hepatic *Zbtb18* could increase the *FXR* mRNA and protein expression through direct binding to the AACTCTCT element on the promoter region of *FXR*. In turn, the *FXR* could stimulate its target genes to accelerate FAO, thereby preventing the onset and development of NAFLD. Moreover, the *Zbtb18/FXR* axis-stimulated *CLTC* protein remarkably alleviates the liver inflammatory infiltrations and fibrosis. Therefore, the *Zbtb18/FXR* axis represents a novel candidate to target for the treatment of NAFLD and NASH.

## Methods

### Clinical tissue preparation

Fifteen liver biopsy samples were taken from patients with preoperative testing at the First Affiliated Hospital of Guangzhou University of Chinese Medicine according to the ethical guidelines of the Declaration of Helsinki. A previous approval was obtained from the Ethics Committee of the First Affiliated Hospital of Guangzhou University of Chinese Medicine (No. K [2023] 013). Written informed consent was obtained from each patient included in the study. Liver samples were divided into the normal group and NAFLD group based on a gold-standard histological classification (i.e., NAFLD activity score [NAS]) and liver pathology.

### Animals and treatments

Male *db/db*, *ob/ob* and their control mice (aged 6–8 weeks) were purchased from GemPharmatech Co., Ltd (Guangdong, China). C57BL/6 J mice aged 6–8 weeks were bought from the Animal Experimental Center of Guangzhou University of Chinese Medicine (Guangdong, China). *Zbtb18* conditional knockout mice were generated by crossing *Alb-Cre* mice with *Zbtb18*^*fl*/fl^ mice. *Zbtb*^*fl*/*fl*^ mice were generated via embryo stem cells (ESCs) targeting technology (Cyagen Inc. Guangzhou, China). In the targeting vector, Exon 2 was selected as the conditional knockout region, the SdNeocassette was flanked by SDA (self-deletion anchor) sites, and the CKO region was flanked by *loxp* sites. Diphtheria toxoid (DTA) was used for the negative selection. The linearized vector was infected into C57BL/6 ESCs. After G418 selection, resistant clones were picked and identified by PCR and Southern blot to choose the correctly targeted ones. Targeted ES cell clones 2D10 and 1E12 were injected into C57BL/6 albino embryos, which were then re-implanted into CD-1 pseudo-pregnant females. Founder animals were identified by their coat color, and their germline transmission was confirmed by breeding with C57BL/6 females and subsequent genotyping of the offspring. The sdNeo cassette served as a self-excision cassette to generate selected-gene-free heterozygous *flox* mice from chimeras. Four female heterozygous targeted mice were generated from clone 2D10, one male and one female heterozygous targeted mouse were generated from clone 1E12, as final deliverables for this project. The genotyping primers were as follows: Forward primer, 5’-TACCTGCAGATCTTACCGC-3’; reverse primer, 5’-CAGGCAAAGTCCCACACAAA-3’. The positive founder mice and wild-type male mice were bred to get F1 *Zbtb18* heterozygote mice.

*Zbtb18* conditional knockin mice were generated by crossing *Alb-Cre* mice and Rosa26-*Zbtb18*^*LSL*^ mice. Rosa26-*Zbtb18*^*LSL*^ mice were generated by Suzhou Cyagen Co., Ltd (Suzhou, China) by applying the CRISPR/Cas9 system. Briefly, for *Zbtb18* knock-in mice, a *CAG-loxp-stop-loxp* sequence followed by mouse *Zbtb18* was inserted at Rosa26 locus (Rosa26-LSL-*Zbtb18*) using the CRISPR/Cas9 system. The sequence of the small guide RNA (sgRNA) was 5′-CTCCAGTCTTTCTAGAAGAT-GGG-3′. *Zbtb18* knock-in mice were mated to *Alb-Cre* mice to generate hepatocyte-specific *Zbtb18* knock-in mice (ROSA26-*Zbtb18*; *Alb-Cre*). *FXR* knockout mice were maintained in our lab as previously reported.^22^ All mice were given free access to food and water and were maintained a 12/12 h light-dark cycle at 25 °C ambiences. HFD 60% (D12492, Research Diets New Brunswick, NJ, USA) was used to establish a DIO mice model and MCD (Trophic Animal Feed High-Tech Co., Ltd, China) for four weeks to establish fibrosis models as previously reported.^46^ Animal care or experimental designs were following the guidelines of and approved by the Animal Ethics Committee of Guangzhou University of Chinese Medicine (approval NO. 20221207001).

Adeno-associated virus expressing *Zbtb18* (AAV-*Zbtb18*), adeno-associated virus expressing green fluorescent protein (AAV-*Gfp*) and all plasmids were constructed and purchased from Hanbio Biotechnology Co. Ltd. (Shanghai, China). A total amount of 0.5–1 × 10^11^ v.g. diluted in 200 µl of PBS was injected into the tail vein of mice 4 weeks before the indicated experiments. Adenovirus expressing green fluorescent protein (Ad-*Gfp*), adenovirus expressing *Zbtb18* (Ad-*Zbtb18*), adenovirus expressing *FXR* (Ad-*FXR*), and all plasmids were constructed and purchased from Shanghai Obio Technology Company (Shanghai, China). 1.0–1.5 × 10^9^ active viral particles in 200 μL saline were injected into the tail vein of mice and 7–9 days after infection, mice were subjected to the designed experiments or fasted for 6 h and their livers and plasma samples were collected for further analysis.

### Histological analysis, Immunofluorescence analysis, and Lipid Droplet staining

H&E and Oil red O staining were conducted as previously reported. [1] Briefly, liver tissues fixed with 10% neutral-buffered formalin were cut into 7 μm-thick sections, followed by routine H&E, Oil Red O, and Sirius staining. Histological analysis was performed by an experienced hepatopathologist from the First Affiliated Hospital of Chinese Medicine using a light microscope (Olympus). Immunofluorescence was conducted at Servicebio (Wuhan, China) according to the previous protocols. Liver sections or MPHs were incubated at 4 °C overnight with primary antibodies against F4/80 (Servicebio, China), CD11b (Servicebio, China), α-SMA (Abmart, China), FXR (Abcam, USA), Bsep (Abmart, China) and *Zbtb18* (Proteintech, China), followed by incubation with indicated secondary antibodies. To detect lipid droplets, liver sections were incubated with BODIPY 493/503 (Thermofisher Scientific) for 30 min. All fluorescence images were obtained using a confocal laser scanning microscope (Leica) or a light microscope (Olympus).

### Fatty acid oxidation detection

The assays were performed as previously described at Soochow Pukang Biotechnology Co., LTD.^35,47^ Briefly, primary hepatocytes infected with the indicated adenovirus were incubated with 0.4 μCi/ml [9,10-^3^H]oleic acid (Perkin Elmer Life Sciences) and 100 μm unlabeled oleic acid (conjugated with BSA) in Krebs-Ringer buffer (119 mM NaCl, 5 mM KCl, 2 mM CaCl_2_, 2.6 mM MgSO_4_, 24.6 mM NaHCO_3_, 2.6 mM KH_2_PO_4_, 10 mM HEPES, pH 7.4) for 3 h at 37 °C. Chloroform was used to extract the unoxidized oleic acid. Supernatants were collected and incubated with 1.3 M perchloric acid. All the solutions were then centrifuged at 16,000 × *g* for 10 min. Supernatants were neutralized with 2 M KOH, 0.6 M MOPS. Next, 3 mL of scintillation liquid was then added and [^3^H] radioactivity was measured.

### Tolerance tests

Tolerance tests were conducted as previously reported. Briefly, for the glucose tolerance test (GTT), mice were fasted overnight and ip. injected with D-glucose (1–2 g/kg), and blood glucose was assessed at 0, 15, 30, 45, 60, 90, and 120 min in the tail vein using a glucose monitor (OnCall EZIV, China). For the insulin tolerance test (ITT), mice were fasted for 6 h and ip. injected with insulin (0.5–0.75 U/kg) followed by the measurement of blood glucose at the same time points.

### Metabolic cages and MRI

To monitor energy expenditure, mice were individually housed and adapted for 24 h in metabolic cages. Their heat production, respiratory exchange ratio (RER), carbon dioxide production (VCO_2_), oxygen consumption (VO_2_) and X-axis ambulation (XAMB) were recorded during the next 36 h by using a Promethion monitoring system (SABLE). Data of 24 h were shown as previously reported. An Echomri.Combo-700 was used to detect the body composition including fat mass and lean mass.

### ELISA and determination of TGs and TCs

Serum ketone levels were assessed using ELISA kits (Ruixinbio, Quanzhou, China). Serum proinflammatory cytokines IL-1β, IL-6, or TNF-α were measured using ELISA kits (Ruixinbio, Quanzhou, China). Serum and hepatic TGs and TCs levels were quantified using reagent kits from Jiancheng Bioengineering Institute (Nanjing, China).

### Luciferase reporter gene assay

HepG2 cells (American Type Culture Collection, Manassas, VA, USA) were cultured in 24-well plates using Dulbecco’s Modified Eagle Medium containing 10% (vol./vol.) FBS (Invitrogen). Luciferase reporter genes were then co-transfected into the cells together with the indicated expression plasmids. The Ramlila luciferase expression vector pCMV-RL-TK (Promega) was used as an internal control. After 48 h, cells were harvested and assessed for luciferase activity using the Dual-Luciferase Reporter Assay System (Promega). Relative luciferase activity was corrected for Renilla-luciferase activity of pCMV-RL-TK and normalized to the activity of the controls. The 5’ end of the mouse *FXR* gene extending from position −1838 bp (relative to the transcription start site) to +47 was cloned into the pGL3-Basic (Promega) luciferase reporter plasmid with the MluI/XhoI sites. A series of 5’ deletions and corresponding mutation constructs of *FXR* (−1074Luc, −677Luc, mut) were prepared by PCR using −1838-Luc as a template. The primers used for plasmid construction are shown in the Supplemental Table.

### Chromatin immunoprecipitation (ChIP) assay

Pulverized liver tissues from C57BL/6 J, HFD, db/m or *db/db* mice were lysed and sonicated. The protein–DNA complexes were immunoprecipitated with mouse IgG antibody (control) or anti-Zbtb18 antibody. The promoter region of *FXR* was amplified by PCR or qPCR using the following primer pair: 5’-TGAGACAGGATTTCTCTATG-3’ as the forward primer and 5’-CCTTTAATCCTAGCACTTA-3’ as the reverse primer.

### RNA-sequencing

RNA sequencing was performed and analyzed at Berry Genomics Corporation (Beijing, China) as previously reported. Briefly, 1 µg RNA isolated from indicated samples were used as input material for the RNA sample preparations. Sequencing was conducted using NEBNext® Ultra™ RNA Library Prep Kit for Illumina® (NEB, USA) according to the manufacturer’s recommendations, followed by adding an Index code in each sample to attribute sequences. A cBot Cluster Generation System was used to perform the clustering of the index-coded samples using TruSeq PE Cluster Kit v3-cBot-HS (Illumina) according to the manufacturer’s instructions. The library preparations were sequenced on an Illumina NovaSeq platform and 150 bp paired-end reads were generated. HTSeq v0.6.1 was used for the subsequent analysis.

### ChIP-sequencing and Peak finding

Input DNA and ChIP DNA were prepared for ChIP-seq via Illumina kit per the manufacturer’s protocol. Briefly, each sample was subjected to end repair, followed by the addition of an A base to the 3′ end. Adaptor ligated DNA fragments were size-selected (175–225 bp), PCR-amplified, and further size-selected (175–225 bp). ChIP-seq data was obtained via Illumina Genome Analyzer II. 36 base pairs were analyzed by Illumina’s pipeline software for quality filtering, and aligned to the Mm9 reference mouse genome. Only uniquely aligned reads were kept for subsequent bioinformatics analysis. To identify *Zbtb18* peaks, 2e analyzed the Chip-seq data by the latest version of MACS2 software.

### Western blot analysis

Protein samples isolated from indicated cells or liver samples were lysed, and homogenized, and their concentration was assessed by using RIPA lysis buffer and a bicinchoninic acid protein assay kit (Beyotime Biotechnology, Shanghai, China). Subsequently, 80–100 μg of protein was loaded onto a 10% SDS–PAGE gel, and the separated proteins were transferred to polyvinylidene difluoride membranes. Specific antibodies were used to conduct western blot assays, with rabbit anti-Zbtb18 from Proteintech (Wuhan, China), anti-FXR, anti-SHP, anti-BSEP, anti-AKT, anti-p-AKT, anti-GSK-3β, anti-p-GSK-3β, anti-NLRP3, anti-ASC, anti-Capase-1, anti-NF-кB and anti-p-NF-кB, which were purchased from Abmart (Shanghai China), and anti-β-actin bought from Abclonal (Wuhan, China) according to their manufacturers’ instructions.

### Real-time quantitative PCR

Total RNA was isolated from indicated cells or liver samples using a Trizol reagent kit (Invitrogen), followed by a reverse-transcription to cDNA using a high-capacity cDNA reverse-transcription kit. Subsequent q-PCR was conducted with the resulting cDNA, using PowerUp^TM^ SYBRTM Green Master Mix, and β-actin was used to normalize the expression of genes. The specific primer sequences are shown in Supplementary Table 1.

### Cell culture and pretreatment

Mouse primary hepatocytes (MPHs) were isolated as previously reported and cultured in a RPMI-1640 medium (10% fetal bovine serum, 100 units/ml penicillin, and 0.1 mg/ml streptomycin). Cells were infected with Ad-*Gfp*, Ad-*Zbtb18* or Ad-*FXR* for 12 h, and next exposed to OA (660 μmol/L) and PA (330 μmol/L) for another 24 h to be thereafter collected for subsequent analysis. BMDM cells were cultured in the cell-conditioned medium collected as previously described.

### Supplementary information

Supplementary Material

## Data Availability

The RNA-sequencing data have been deposited in NCBI under SRA accession numbers (Zbtb18 overexpressing MPHs: PRJNA994862; Clinical NAFLD patients: PRJNA994268). ChIP-seq data have been deposited in NCBI under SRA accession numbers (SRA: PRJNA998803). All data in this article are available upon reasonable request from the corresponding authors.
