# Supplementary material for: Hepatic Zbtb18 (Zinc Finger and BTB Domain Containing 18) alleviates hepatic steatohepatitis via FXR (Farnesoid X Receptor)
Source: Signal Transduct Target Ther. 2024 Jan 24;9:20. doi: 10.1038/s41392-023-01727-7 (PMC10806020; doi:10.1038/s41392-023-01727-7)
Supplement: Supplementary file 1 — Supplementary Material [file 41392_2023_1727_MOESM1_ESM.docx]

Supplementary Materials for

Hepatic *Zbtb18* (Zinc Finger and BTB Domain Containing 18) alleviates hepatic steatohepatitis via *FXR* (Farnesoid X Receptor)

Lei Zhang^1,2#^, Jiabing Chen^1#^, Xiaoying Yang^3#^, Chuangpeng Shen^4#^, Jiawen Huang^1#^, Dong Zhang^5^, Naihua Liu^1^, Chaonan Liu^4^, Yadi Zhong^1^, Yingjian Chen^1^, Kaijia Tang^1^, Jingyi Guo^1^, Tianqi Cui^1^, Siwei Duan^1^, Jiayu Li^1^, Shangyi Huang^1^, Huafeng Pan^1,8^, Huabing Zhang^6^, Xiaoqiang Tang^7*^, Yongsheng Chang^2*^, Yong Gao^1,8*^

#These authors made equal contributions to this work.

Correspondence to: [gaoyong@gzucm.edu.cn](mailto:gaoyong@gzucm.edu.cn) (Y.G.), [changys@tmu.edu.cn](mailto:changys@tmu.edu.cn) (YS.C.), tangxiaoqiang@scu.edu.cn (XQ.T.).

**This PDF file includes:**

Materials and Methods

Supplementary Figures. S1 to S11

Supplementary Tables. 1 to 2

**Supplementary Materials and Methods**

Proteomics analysis

The conditional medium of Ad-*Gfp-* or Ad-*Zbtb18*-infected samples was subjected to proteomic analysis at Guangzhou Kefu-tech (Guangzhou, China). Briefly, the tryptic digests were analyzed by a Q Exactive plus in positive mode. All MS and MS/MS spectra were obtained in data-dependent mode with one MS full-scan ranging from 300 to 1800 followed by 10 MS/MS scans. The raw files obtained were up-loaded to Proteome Discoverer (PD, version 2.1) and searched against the UniProtKB mouse complete proteome sequence database (release 2019_04; 85,121 entries) to show the modification of peptides identified. The parameters for searching were set as follows: enzyme, trypsin; missed cleavages, two; fixed modifications, carboxyamidomethylation (C); variable modifications, oxidation (M) and acetylation protein N-termini. The peptide tolerance and MS/MS tolerance were 10 ppm and 0.02 Da, respectively. The ascertained false discovery rate (FDR) for all the identified peptides was less than 1%.

Statistical analyses

Data were shown as means ± SEM and to assess the significant differences among groups Student’s t test or one-way ANOVA followed by LSD or Dunnett’s post-hoc tests were used. *P* < 0.05 was considered statistically significant.

**Supplementary Figures**


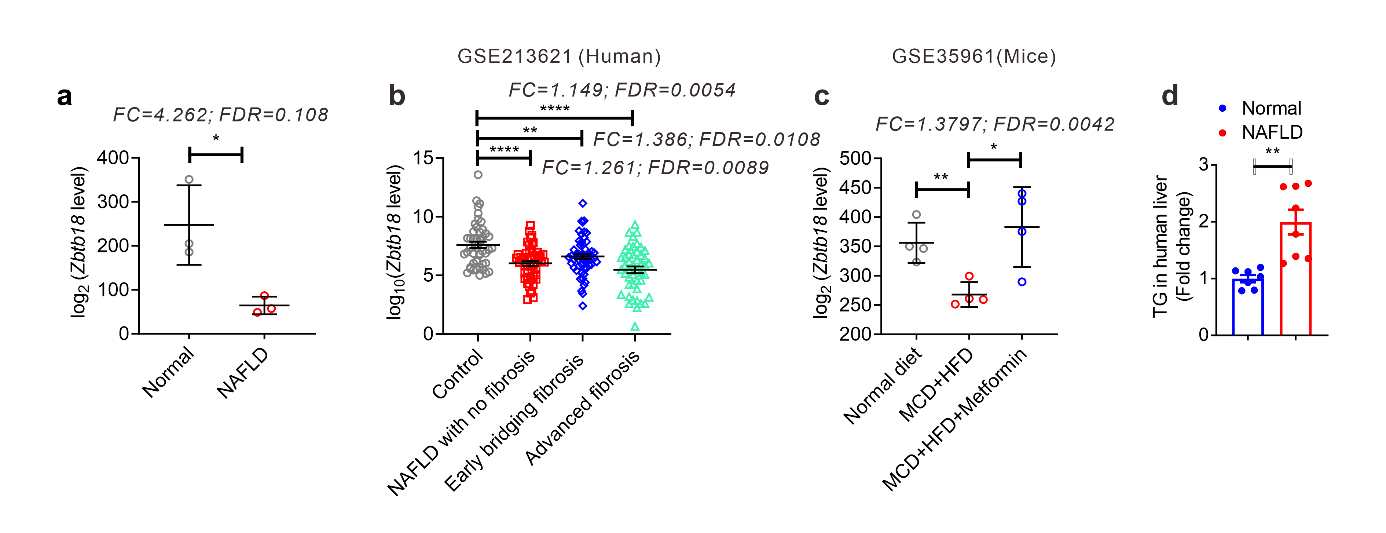


**Fig. S1 Hepatic *Zbtb18* expression is significantly decreased in NAFLD patients.**

(a-c) The mRNA levels of *Zbtb18* in NAFLD patients (a) and GSE213621 (b), and GSE35961 (c) data sets. (d) TG contents of normal and NAFLD clinical samples. Data are shown as means ± SEM. **P*<0.05; ***P*<0.01; *****P*<0.001.


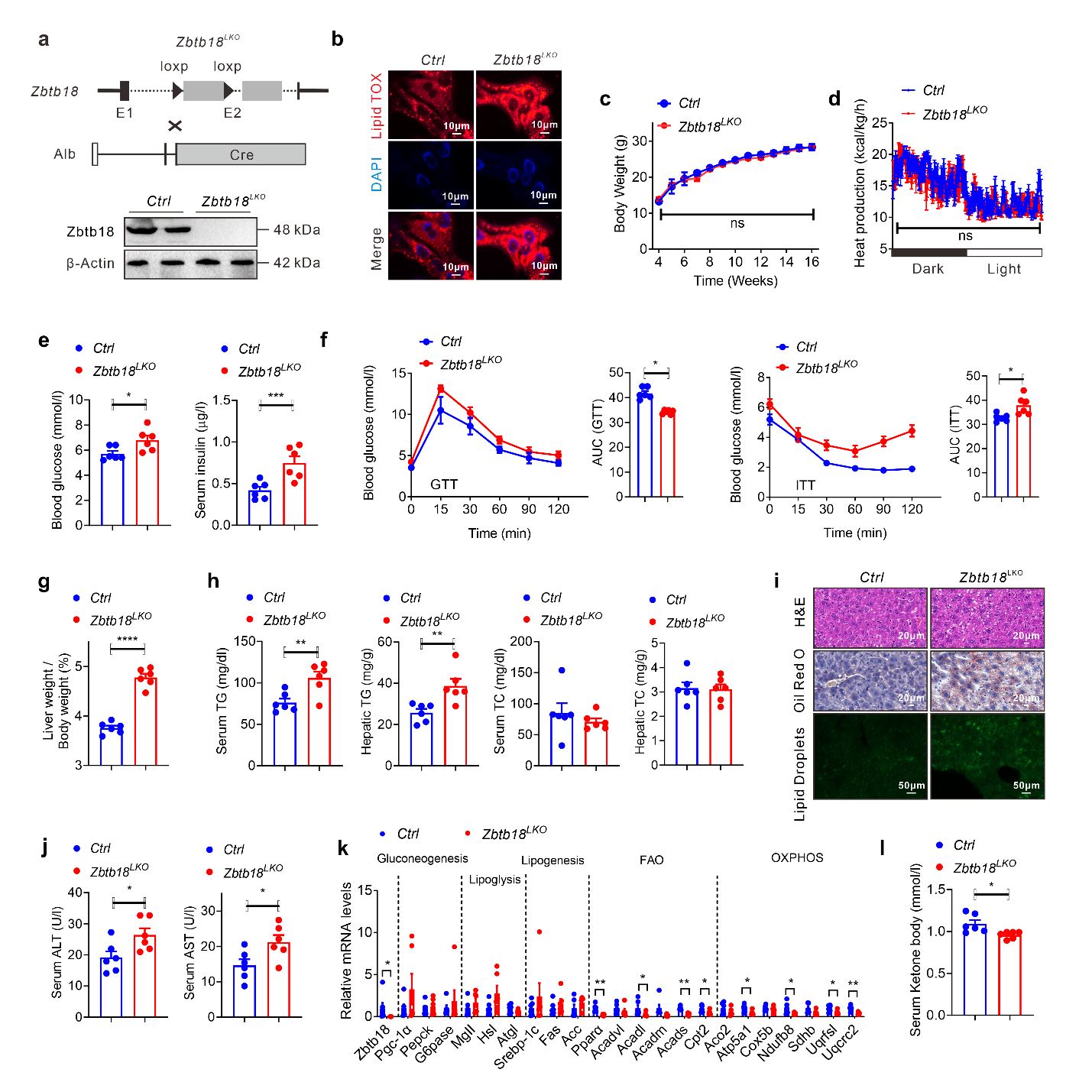


**Fig. S2 Hepatic ablation of *Zbtb18* makes mice fed on a chow diet more prone to steatohepatitis**

(a) Upper, the schematic diagram depicting the construction of *Zbtb18^LKO^* mice. Bottom, Western blot analysis proved the deletion of hepatic *Zbtb18* in mice. (b) Deletion of *Zbtb18* aggravates the OA&PA-induced lipid accumulation in cultured MPHs. (c-d) Hepatic *Zbtb18* deletion doesn’t change the body weight (c) and body heat production (d) of mice fed on a chow diet; n≥6. (e) Hepatic *Zbtb18* deletion increases the fasting blood glucose and insulin levels in mice fed on a chow diet; n=6. (f) Hepatic *Zbtb18* deletion impairs the glucose, and insulin sensitivity of mice fed on chow diet; n=6. (g-h) Hepatic *Zbtb18* deficiency increases the ratio of liver weight to body weight (g) and TG & TC contents in serum and liver (h) samples of mice fed on chow diet; n=6. (i) H&E, Oil red O staining, and BODIPY 493/503 staining indicate that hepatic *Zbtb18* deletion leads to lipid accumulation in the liver of mice fed on a chow diet. (j-k) Hepatic *Zbtb18* deficiency decreases serum ALT and AST levels in mice fed on chow diet; n=6 (j) and alters the expression of hepatic genes related to glucose and lipid metabolism (k) in mice fed on a chow diet; n≥4. (l) Hepatic *Zbtb18* deficiency decreases serum ketone body levels; n=6. Data are shown as means ± SEM. **P*<0.05; ***P*<0.01; ****P*<0.005; *****P*<0.001.


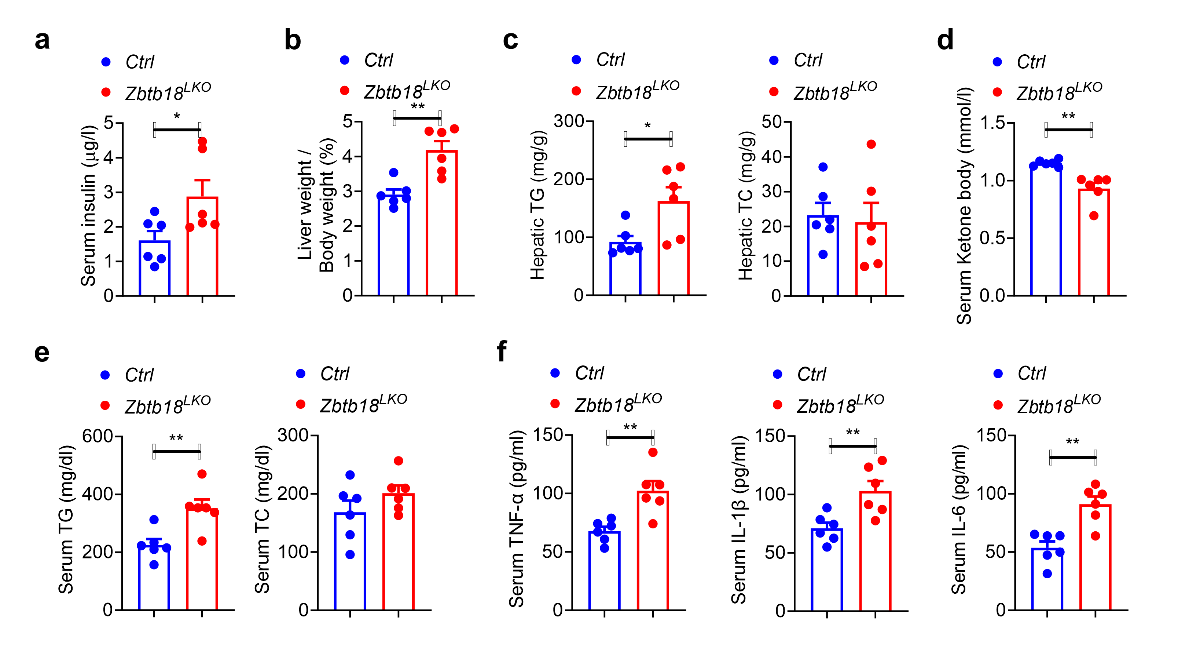


**Fig. S3 Hepatic *Zbtb18* ablation makes mice more prone to steatohepatitis.**

(a) Hepatic *Zbtb18* ablation increased the serum insulin levels of mice fed on HFD; n=6. (b) Hepatic *Zbtb18* deletion increased the ratio values of liver weight/body weight in the mice fed on HFD; n=6. (c) Hepatic *Zbtb18* deletion increased the TG & TC contents in liver samples while having no effects on TC levels; n=6. (d) Hepatic *Zbtb18* deletion reduced the serum ketone body levels in the mice fed on HFD; n=6. (e) Hepatic *Zbtb18* deletion increased the TG contents in serum, while having no effects on TC levels; n=6. (f) Hepatic *Zbtb18* deletion significantly increased the levels of serum proinflammatory cytokines of mice fed on HFD; n=6. Data are shown as means ± SEM. **P*<0.05; ***P*<0.01.


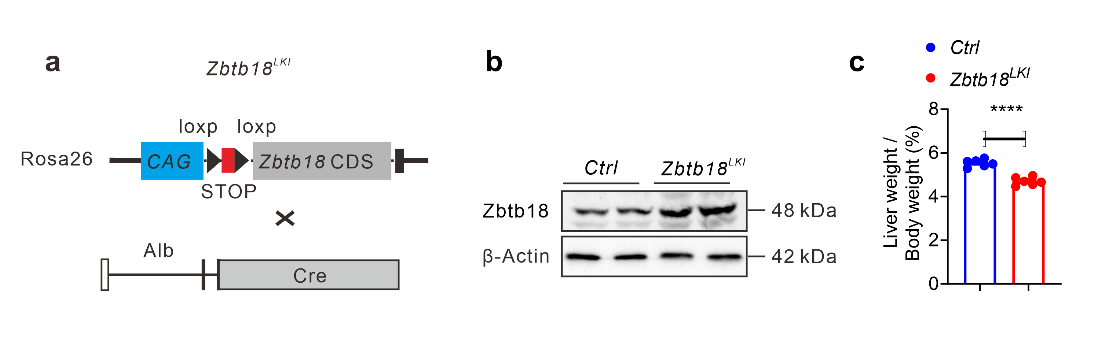


**Fig. S4 Hepatic** ***Zbtb18***, **overexpression mitigates HFD-induced hepatosteatosis.**

(a) The schematic diagram depicting the construction of *Zbtb18^LKI^* mice. (b) Western blot analysis proved the increased expression of *Zbtb18* protein in hepatic *Zbtb18* transgenic mice. (c) Hepatic *Zbtb18* overexpression decreased the ratio values of liver weight relative to body weight in mice fed on HFD; n=6. Data are shown as means ± SEM. ns= no significant; *****P* < 0.001.


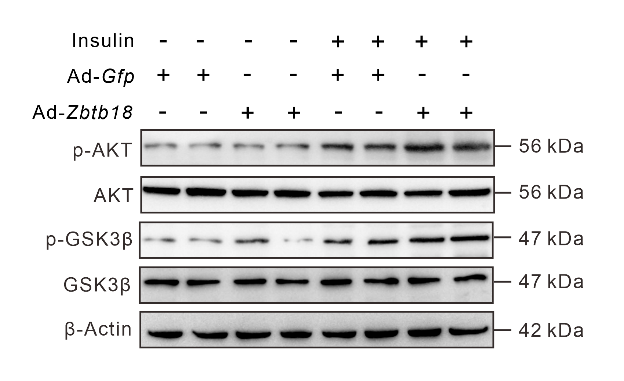


**Fig. S5** **Rescued *Zbtb18* expression increases the phosphorylation of AKT and GSK-3β in MPHs.** Western analysis phosphorylation levels of AKT and GSK-3β in MPHs.


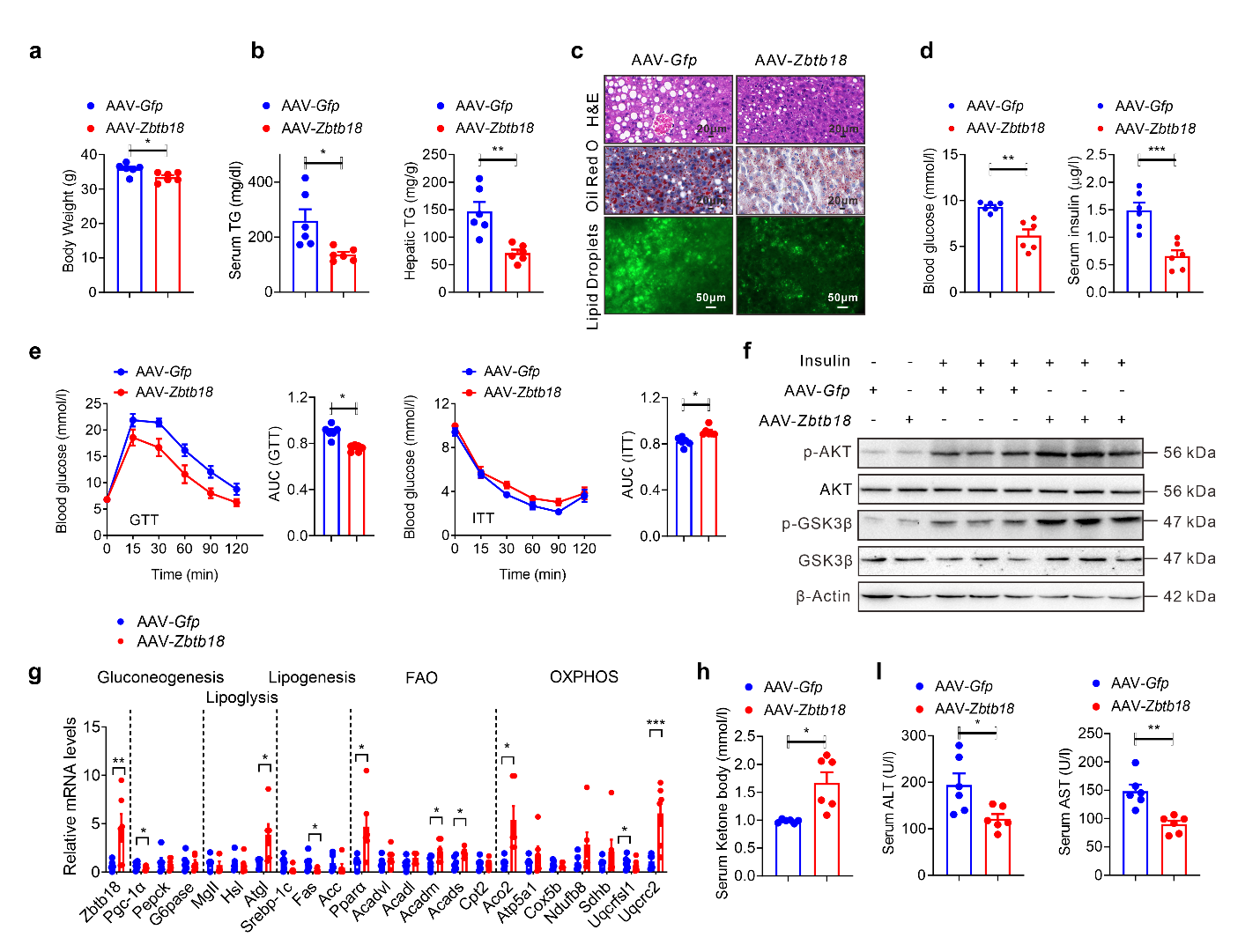


**Fig. S6 Rescued *Zbtb18* expression protects against HFD-induced liver steatosis in hepatic *Zbtb18* deficient mice**

(a-b) Hepatic *Zbtb18* deficient mice infected with AAV-*Zbtb18* showed decreased body weights (a) and TG contents in liver and serum samples (b), even when fed on HFD; n=6. (c) AAV-*Zbtb18* infection improved hepatic lipid accumulation in hepatic *Zbtb18* deficient mice fed on HFD. (d-e) Rescued *Zbtb18* expression in livers decreased the fasting blood glucose and insulin levels (d), and improved glucose tolerance and insulin sensitivity (e) in hepatic *Zbtb18* deficient mice fed on HFD; n=6. (f) Increased liver *Zbtb18* expression enhanced the phosphorylation of AKT and GSK-3β in hepatic *Zbtb18* deficient mice fed on HFD. (g-h) AAV-*Zbtb18* infection altered the expression of hepatic genes related to glucose and lipid metabolism (g) and elevated serum ketone body levels (h) in hepatic *Zbtb18* deficient mice fed on HFD; n=6. (i) AAV-*Zbtb18* infection decreased serum ALT and AST levels in hepatic *Zbtb18* deficient mice fed on HFD; n=6. Data are shown as means ± SEM. ns= no significant; **P*<0.05; ***P*<0.01; ****P*<0.005.


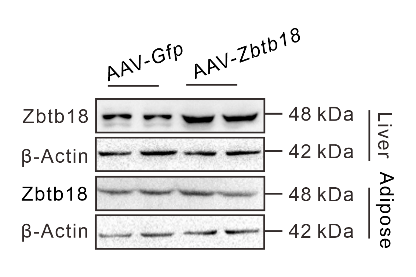


**Fig. S7 Hepatic *Zbtb18* expression in the liver of db/db mice following AAV-*Zbtb18* infection.**

Western blot data indicated the specific overexpression of Zbtb18 protein in the liver of db/db mice following AAV-*Zbtb18* infection.


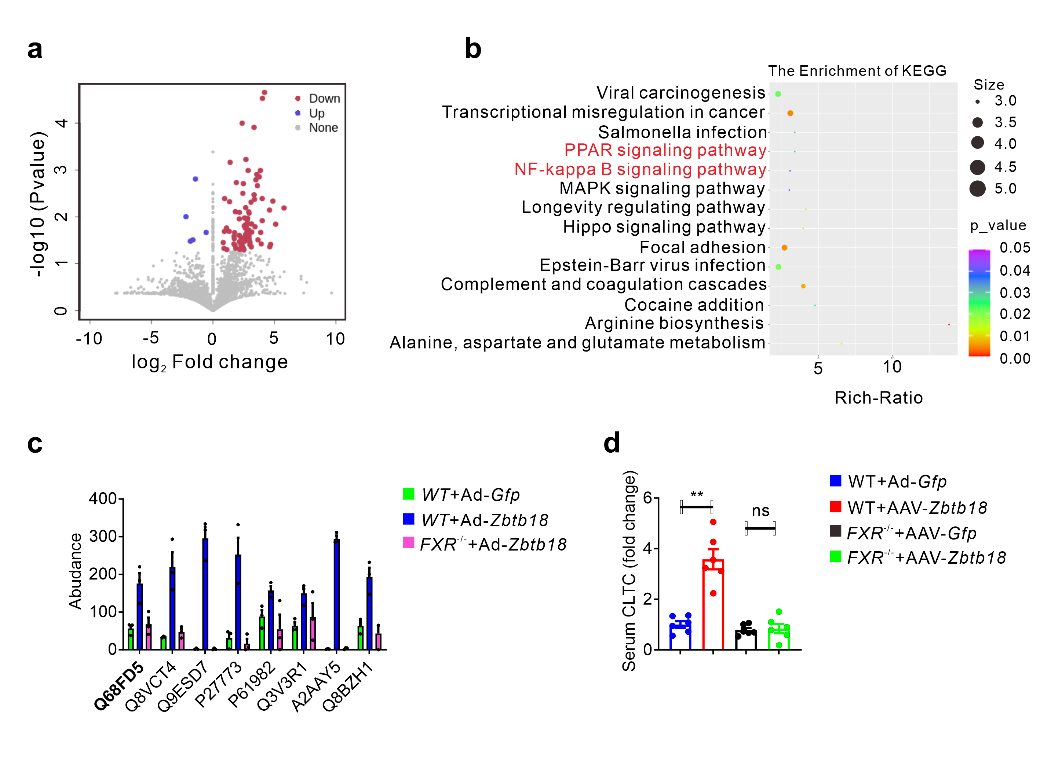


**Fig. S8 Hepatic *Zbtb18*** **expression inhibited NLRP3 activation in macrophages via the *FXR*-mediated CLTC protein secretion**

(a-b) Proteomic analysis of the supernatants of Ad-*Zbtb18* infected hepatocytes indicated that *Zbtb18* overexpression significantly increased the abundance of proteins related to the inflammatory response, as shown by Scatter plot (a), and KEGG analysis (b). (c) *Zbtb18* expression increased the CLTC protein from hepatocytes via *FXR*; n=3. (d) *FXR* deletion diminished the *Zbtb18*-stimulated upregulation of CLTC in mice; n=6. Data are shown as means ± SEM. ns= no significant; ***P*<0.01.


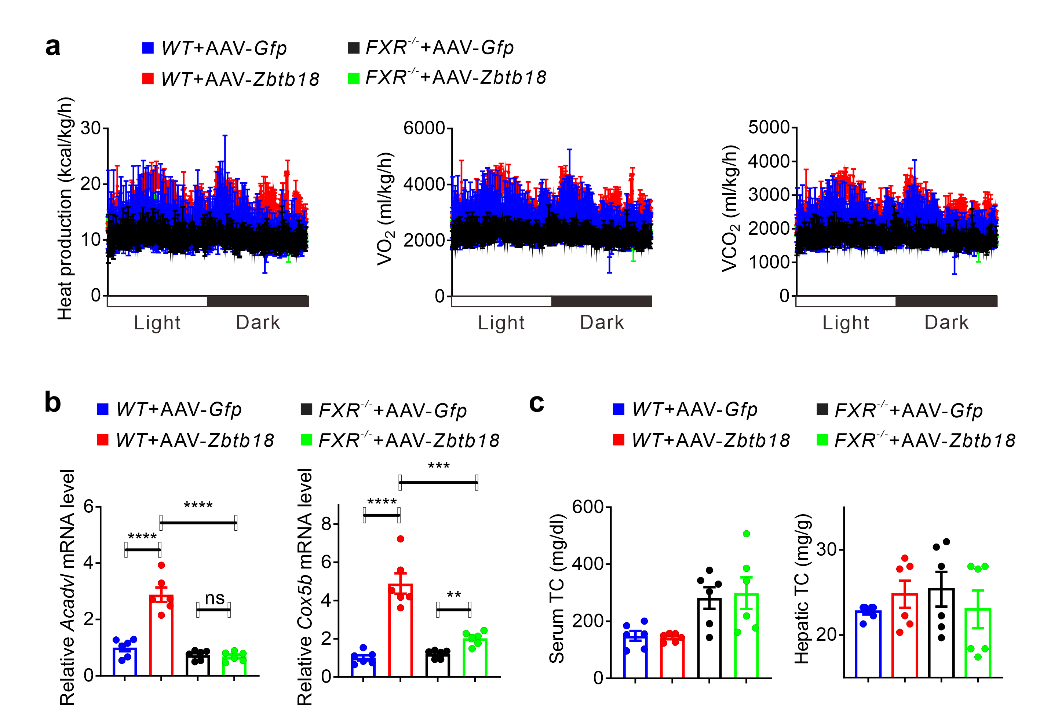


**Fig. S9 *FXR* ablation diminished the hepatic *Zbtb18*-induced protective effects against steatohepatitis**

(a) The VO_2_ consumption, VCO_2_ production and heat production of AAV- *Zbtb18-* or AAV-*Gfp*-infected *FXR* knockout mice and control mice fed on HFD; n≥4. (b) *FXR* deletion abolished *Zbtb18*-stimulated expression of genes related to fatty acid oxidation; n=6. (c) The TC contents in serum and liver samples from AAV-*Zbtb18* or AAV-*Gfp* infected *FXR* knockout mice and control mice fed on HFD; n=6. Data are shown as means ± SEM. ns= no significant; ***P*<0.01; ****P*<0.005; *****P*<0.001.


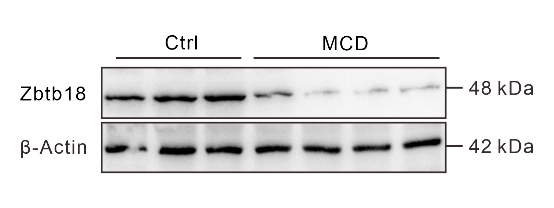


**Fig. S10 Hepatic *Zbtb18* protein protects mice against MCD-induced liver fibrosis.**

Western blot data indicated the expression of *Zbtb18* protein was decreased after MCD exposure.


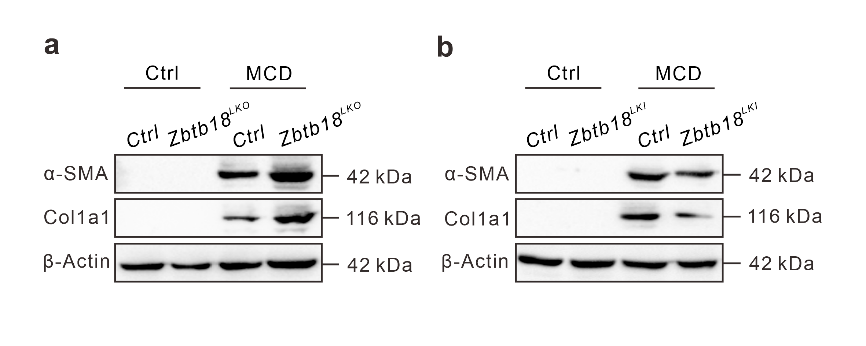


**Fig. S11 Hepatic *Zbtb18* protein protects mice against MCD-induced liver fibrosis.**

(a-b) Western blot data indicated the expression of α-SMA and Col1a1 protein was regulated by *Zbtb18* after MCD exposure.

| **Patient** | **Gender** | **Age** | **Diagnosis** | **Blood glucose (mmol/L)** | **TG (mmol/L)** | **TC (mmol/L)** |
| --- | --- | --- | --- | --- | --- | --- |
| NAFLD 1 | Female | 56 | Moderate fatty liver,  Type 2 diabetes | 8.17 | 4.72 | 3.79 |
| NAFLD 2 | Male | 53 | Mild fatty liver,  Type 2 diabetes | 10.28 | 3.07 | 3.83 |
| NAFLD 3 | Female | 46 | Severe fatty liver,  Type 2 diabetes | 8.32 | 3.25 | 3.85 |
| NAFLD 4 | Male | 42 | Severe fatty liver,  Type 2 diabetes | 8.88 | 9.19 | 7.72 |
| NAFLD 5 | Male | 58 | Severe fatty liver,  Type 2 diabetes | 7.37 | 4.24 | 6.02 |
| NAFLD 6 | Male | 55 | Mild fatty liver,  Type 2 diabetes | 11.56 | 12.65 | 4.24 |
| NAFLD 7 | Male | 45 | Type 2 diabetes | 7.13 | 3.51 | 4.52 |
| NAFLD 8 | Male | 57 | Mild fatty liver,  Type 2 diabetes | 11.11 | 6.64 | 3.51 |
| Non-NAFLD 1 | Male | 29 | N/A | 3.61 | 0.96 | 3.35 |
| Non-NAFLD 2 | Female | 31 | N/A | 5.24 | 1.34 | 2.94 |
| Non-NAFLD 3 | Male | 64 | N/A | 3.94 | 0.99 | 5.6 |
| Non-NAFLD 4 | Female | 64 | N/A | 4.56 | 1.51 | 5 |
| Non-NAFLD 5 | Male | 38 | N/A | 3.87 | 1.62 | 4.43 |
| Non-NAFLD 6 | Male | 59 | N/A | 5.02 | 0.92 | 2.76 |
| Non-NAFLD 7 | Male | 64 | N/A | 5.38 | 0.71 | 4.45 |

**Table S1. Clinical characteristics of Non-NAFLD and NAFLD patients**

| **Primer** | **Sequences** |
| --- | --- |
| *Zbtb18* (human) | F1: TCTGAGCGAGCAGAGACAC  R1: GGTCCTTGTAAAAGAGGTGGAAA |
| *Zbtb18* (mouse) | F1: CTGAACAGCGACATTGTGACA  R1: GCTCTCGACTTTATCCGAACAAC |
| *Srebp-1c* (mouse) | F1: GGAGCCATGGATTGCACATT  R1: GGCCAGGGAAGTCACTGT |
| *Pgc-1α* (mouse) | F1: TATGGAGTGACATAGAGTGTGCT  R1: GTCGCTACACCACTTCAATCC |
| *Pepck* (mouse) | F1: CTGCATAACGGTCTGGACTTC  R1: GCCTTCCACGAACTTCCTCAC |
| *G6pase* (mouse) | F1: CGACTCGCTATCTCCAAGTGA  R1: GGGCGTTGTCCAAACAGAAT |
| *β-actin* (mouse) | F1: CTCCAGAACGCAAGTACTCT  R1: CCAGCTTCGTCGTATTCCTG |
| *Fas* (mouse) | F1: CTTGGGTGCTGACTACAACC  R1: GCCCTCCCGTACACTCACTC |
| *Acc* (mouse) | F1: AGGAAGATGGCGTCCGCTCTG  R1: GGTGAGATGTGCTGGGTCAT |
| *Pparα* (mouse) | F1: AACATCGAGTGTCGAATATGTGG  R1: CCGAATAGTTCGCCGAAAGAA |
| *MgII* (mouse) | F1: AGGCGAACTCCACAGAATGTT  R1: ACAAAAGAGGTACTGTCCGTCT |
| *Hsl* (mouse) | F1: GATTTACGCACGATGACACAGT  R1: ACCTGCAAAGACATTAGACAGC |
| *Atgl* (mouse) | F1: ATGTTCCCGAGGGAGACCAA  R1: GAGGCTCCGTAGATGTGAGTG |
| *Acadvl* (mouse) | F1: ACTACTGTGCTTCAGGGACAA  R1: GCAAAGGACTTCGATTCTGCC |
| *Acadl* (mouse) | F1: TTTCCTCGGAGCATGACATTTT  R1: GCCAGCTTTTTCCCAGACCT |
| *Acads* (mouse) | F1: GACTGGCGACGGTTACACA  R1: GGCAAAGTCACGGCATGTC |
| *Cpt2* (mouse) | F1: CAGCACAGCATCGTACCCA  R1: TCCCAATGCCGTTCTCAAAAT |
| *Aco2* (mouse) | F1: ATCGAGCGGGGAAAGACATAC  R1: TGATGGTACAGCCACCTTAGG |
| *Atp5a1* (mouse) | F1: TCTCCATGCCTCTAACACTCG  R1: CCAGGTCAACAGACGTGTCAG |
| *Cox5b* (mouse) | F1: GCTGCATCTGTGAAGAGGACAAC  R1: CAGCTTGTAATGGGTTCCACAGT |
| *Ndufb8* (mouse) | F1: TGTTGCCGGGGTCATATCCTA  R1: AGCATCGGGTAGTCGCCATA |
| *Sdhb* (mouse) | F1: CTGAATAAGTGCGGACCTATGG  R1: AGTATTGCCTCCGTTGATGTTC |
| *Uqrfs1* (mouse) | F1: GGTAACTGCAACTACTACTGTGG  R1: CTTGATCTCGATCTTCGACATGG |
| *Uqcrc2* (mouse) | F1: AAAGTTGCCCCGAAGGTTAAA  R1: GAGCATAGTTTTCCAGAGAAGCA |
| *FXR* (mouse) | F1: GGCAGAATCTGGATTTGGAATCG  R1: GCCCAGGTTGGAATAGTAAGACG |
| *Bsep* (mouse) | F1: TCTGACTCAGTGATTCTTCGCA  R1: CCCATAAACATCAGCCAGTTGT |
| *Shp* (mouse) | F1: CAGGTCGTCCGACTATTCTGT  R1: AGGCTACTGTCTTGGCTAGGA |
| *Tnfα* (mouse) | F1: CAGGCGGTGCCTATGTCTC  R1: CGATCACCCCGAAGTTCAGTAG |
| *Il6* (mouse) | F1: CTGCAAGAGACTTCCATCCAG  R1: AGTGGTATAGACAGGTCTGTTGG |
| *Il1β* (mouse) | F1: GAAATGCCACCTTTTGACAGTG  R1: TGGATGCTCTCATCAGGACAG |
| α*-*SMA (mouse) | F1: GCAGGGAGTAATGGTTGGAAT  R1: TCTCAAACATAATCTGGGTCA |
| *Col1a1* (mouse) | F1: CATGAGCCGAAGCTAACCC  R1: TGTGGCAGATACAGATCAAGC |
| *Tgfβ* (mouse) | F1: CCACCTGCAAGACCATCGAC  R1: CTGGCGAGCCTTAGTTTGGAC |
| *Cxcl1* (mouse) | F1: ACTGCACCCAAACCGAAGTC  R1: TGGGGACACCTTTTAGCATCTT |
| *Cxcl10* (mouse) | F1: CCAAGTGCTGCCGTCATTTTC  R1: GGCTCGCAGGGATGATTTCAA |
| *GAPDH* (human) | F1: ACAACTTTGGTATCGTGGAAGG  R1: GCCATCACGCCACAGTTTC |

**Table 2. Primer information for gene amplification.**
